# Perioperative quality indicators among neurosurgery patients: A retrospective cohort study of 1142 cases at a tertiary center

**DOI:** 10.1371/journal.pone.0297167

**Published:** 2024-02-06

**Authors:** Edzhem Chavush, Karl Rössler, Christian Dorfer

**Affiliations:** Department of Neurosurgery, Medical University of Vienna, Vienna, Austria; Columbia University Vagelos College of Physicians and Surgeons, UNITED STATES

## Abstract

**Objective:**

The purpose of this study was to present the first comprehensive analysis of perioperative quality indicators; length of hospital stay; readmission; reoperation; pre-, intra, and postoperative events; and mortality in a diverse neurosurgical patient cohort in Europe.

**Methods:**

Electronic medical records of all patients who were admitted to our institution between January 1 and December 31 of 2020, and underwent an index neurosurgical operation (n = 1142) were retrospectively reviewed.

**Results:**

The median length of hospital stay at the index admission and readmission was 8 days (range: 1–242 days) and 5 days (range: 0–94 days), respectively. Of the 1142 patients, 22.9% (n = 262) had an extended length of hospital stay of ≥14 days. The all-cause 7-, 15-, 30-, 60-, and 90-day readmission rates were 3.9% (n = 44), 5.7% (n = 65), 8.8% (n = 100), 12.3% (n = 141), and 16.5% (n = 188), respectively. The main reason for unplanned readmission was deterioration of medical and/or neurological condition. The all-cause 7-, 15-, 30-, 60-, and 90-day reoperation rates were 11.1% (n = 127), 13.8% (n = 158), 16.5% (n = 189), 18.7% (n = 213), and 19.4% (n = 221), respectively. Unplanned reoperations were due primarily to hydrocephalus. The rate of preoperative events was 1.1% (n = 13), one-third of which were associated with infection. The rate of intraoperative events was 11.0% (n = 126), of which 98 (64.47%) were surgical, 37 (24.34%) were anesthesiologic, and 17 (11.18%) were associated with technical equipment. The rate of postoperative events was 9.5% (n = 109). The most common postoperative event was malfunction, disconnection, or dislocation of an implanted device (n = 24, 17.91%). The mortality rates within 7, 15, 30, 60, and 90 days after the index operation were 0.9% (n = 10), 1.8% (n = 21), 2.5% (n = 29), 3.4% (n = 39), and 4.7% (n = 54), respectively. Several patient characteristics and perioperative factors were significantly associated with outcome parameters.

**Conclusions:**

This study provides an in-depth analysis of quality indicators in neurosurgery, highlighting a variety of inherent and modifiable factors influencing patient outcomes.

## Introduction

In recent years, growing emphasis has been placed on value-based healthcare rather than volume-based healthcare. Advancing the efficiency of healthcare delivery by improving patient outcomes relative to costs not only benefits patients but also increases the economic sustainability of healthcare systems [1]. Accordingly, parameters assessing patient outcomes, such as the length of hospital stay (LOS), readmission rate, reoperation rate, adverse event rate, and mortality rate, are increasingly used as quality metrics [2–8].

The average LOS varies greatly among countries and healthcare systems, and this variation cannot be explained by medical reasons or necessity [9, 10]. As reported by the Organisation for Economic Co-operation and Development, the average LOS in Austria is longer than that in Anglo-American and Scandinavian countries, but shorter than that in Germany [11]. Decreasing the LOS in German-speaking countries is important, because research has suggested that each day spent in the hospital confers additional risks on patients regardless of their primary diagnoses [12]. Hospital-acquired conditions such as air embolism, blood incompatibility, catheter-associated infections, poor glycemic control, and surgical site infections continue to markedly threaten patient safety [13].

Hospital readmission rates can indicate substandard care and unfavorable patient outcomes [4, 14, 15]. As a quality parameter, they indicate the effect of hospital care on patient condition after discharge [16]. Rehospitalization occurs frequently and may continue to be life-threatening [17]. Because inpatient acute care is the most expensive type of health service, hospital readmission results in substantial healthcare costs [16]. Research has suggested that a sizable proportion of readmissions may potentially be avoidable [14].

Reoperation rates are increasing being used as another quality indicator, because of their wide applicability and straightforward determination [18]. According to a study in five European hospitals, 1 minute of operating room time costs approximately 9.45 € in a conventional surgical theater [19]. In hybrid operating rooms, which are also frequently used for neurosurgical procedures, the average cost is more than twice as high, at 19.88 € [19]. Given that neurosurgical operations generally have longer durations than operations in other specialties, unplanned returns to the operating room can greatly increase the overall costs of patient care [20].

Adverse events after neurosurgical procedures are not rare [7]. These events not only endanger patients’ health status, but are also associated with extended LOS, readmission, and reoperation, thereby resulting in substantial healthcare costs [5, 7, 21]. The incidence of adverse events after surgical operations is expected to rise even further, as a consequence of aging of the population and increased burden of illness worldwide [21]. In previous studies, prolonged length of preoperative hospital stay has been identified as a modifiable risk factor for various postoperative adverse events [22–25]. However, an overview of in-hospital complications between hospital admission and index operation is currently not available. Moreover, the overall incidence rate of intraoperative complications among neurosurgery patients has not been investigated in the literature to date. Earlier work in this area was either procedure specific or focused on only pediatric patients. Moreover, no report to date has described the predictive factors associated with intraoperative complications in neurosurgery [26–30].

Therefore, the aim of this study was to present what is, to our knowledge, the first analysis of perioperative quality indicators in a diverse neurosurgical patient cohort in a European healthcare system.

## Materials and methods

### Design and setting

This study was a retrospective cohort analysis of patients at the Department of Neurosurgery of the Vienna General Hospital, Medical University of Vienna. Approval was granted by the ethics committee of the Medical University of Vienna (number 1360/2021), in accordance with the issued Guidelines for Good Scientific Practice. The requirement for informed patient consent was waived by the ethics committee. The Vienna General Hospital is a tertiary referral center, and the largest hospital in Austria and fifth largest hospital in Europe. It has a capacity of 1732 beds [31], and more than 61000 inpatient admissions and more than 1.1 million outpatient visits per year [32].

Electronic medical records of all patients (n = 1142) who were admitted to our institution between January 1 and December 31 of 2020, and underwent an index neurosurgical operation were retrospectively reviewed. Data collection was performed through the patient information management system (AKIM) of Vienna General Hospital by the first author between June 1, 2021, and July 20, 2022. Only the first author had access to information that could be used to identify individual participants during or after data collection.

### Outcome variables

The primary outcome measures of interest were the LOS at the index admission and readmission; rates of all-cause and unplanned readmission within 7, 15, 30, 60, and 90 days after discharge from the index admission; causes of readmission; International Classification of Diseases 10th Version (ICD-10) diagnosis codes at readmission; rates of all-cause and unplanned reoperation within 7, 15, 30, 60, and 90 days after the index operation; ICD-10 diagnosis codes at reoperation; procedures performed at reoperation; causes of reoperation; pre-, intra-, and postoperative events and their rates; time until adverse events; and mortality rates within 7, 15, 30, 60, and 90 days after the index operation.

### Definitions of outcomes

In this study, the LOS spanned from hospital admission to discharge. Extended LOS was defined as a hospital stay exceeding the 75th percentile for the overall cohort (≥14 days). Index hospital admission refers to the first hospital admission of patients to the Department of Neurosurgery of the Vienna General Hospital, in which neurosurgical operation was performed. The index operation was defined as the first neurosurgical operation performed at the Department of Neurosurgery of the Vienna General Hospital. Readmission refers to return to the Vienna General Hospital for any cause after prior discharge. Reoperation describes a repeat neurosurgical procedure after the index surgery. Preoperative events were defined as any adverse event that occurred in the hospital between the index admission and the index operation. Intraoperative events describe any adverse events occurring in the operating theater during the index surgery, as described by the operating surgeon. Postoperative events were defined as any abnormal or unexpected neurosurgical events occurring within 90 days after the index surgery, and contributing to prolonged hospitalization, readmission, or reoperation. Detailed definitions of postoperative events are provided in Table 10 in the S1 Appendix.

### Statistical analysis

SPSS® statistics software, version 29 (IBM Corporation), was used for statistical analysis. Descriptive statistics, including means, medians, rates, ranges, percentages, and standard deviations, was used to describe the collected patient datasets. Univariate analyses between categorical variables were performed with Pearson’s χ2 test, Fisher’s exact test, the Fisher-Freeman-Halton exact test, or logistic regression, as appropriate. A Monte Carlo simulation was applied, if necessary, with a confidence level of 99% and 10000 samples. Continuous variables were compared via Mann-Whitney U test or independent-samples Kruskal-Wallis test with Dunn-Bonferroni post hoc analysis. Multivariate logistic regression models were built with an entry criterion of *p*<0.05 to further investigate the independent predictors of extended LOS, unplanned readmission, unplanned reoperation, perioperative events, and mortality. The Hosmer–Lemeshow test was used to determine the calibration of the models. A *p*-value less than 0.05 was defined as statistically significant. All reported *p*-values are two-sided.

## Results

**Fig 1:**
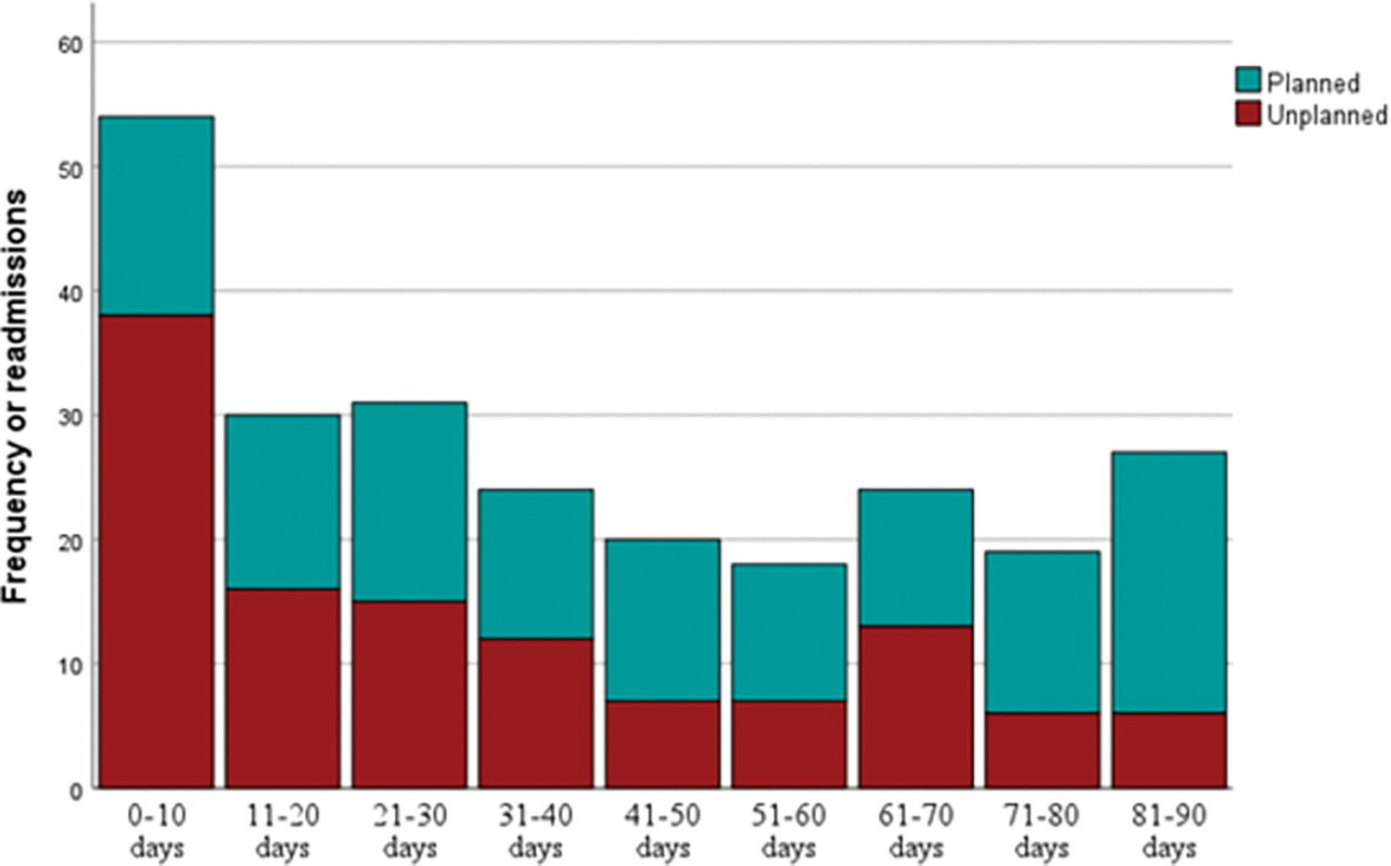
Time until readmission after discharge from hospital.

The baseline characteristics of the 1142 patients in the study population are presented in Table 1. Fig 1 in the S1 Appendix shows the distribution of study participants in cohorts.

**Table 1:** Patient characteristics, clinical features, and outcomes.

	No. of patients Total = 1142		LOS at Index Admission (in days)	Proportion of Patients with an Extended LOS		90-Day Unplanned Readmission Rate		90-Day Unplanned Reoperation Rate		Preoperative Event Rate		Intraoperative Event Rate		Postoperative Event Rate		90-Day Postoperative Mortality Rate	
Variables	N	%	Median	%	*p*-value	%	*p*-value	%	*p*-value	%	*p*-value	%	*p*-value	%	*p*-value	%	*p*-value
Age group (years)					<0.001		0.296		0.046		0.313		0.365		0.702		<0.001
<18	162	14.2	10.0	35.2		13.0		16.0		3.1		11.7		9.3		2.5	
18–29	65	5.7	8.0	13.8		7.7		10.8		1.5		3.1		12.3		1.5	
30–39	130	11.4	7.0	10.0		6.9		5.4		0.0		10.0		8.5		0.8	
40–49	155	13.6	7.0	18.1		5.8		7.7		0.6		9.7		7.1		3.2	
50–59	238	20.8	9.0	23.9		8.8		11.8		0.8		11.8		11.3		5.9	
60–69	192	16.8	9.0	22.4		7.3		11.5		0.5		13.0		11.5		6.3	
70–79	161	14.1	9.0	28.6		7.5		9.9		1.9		12.4		6.8		5.6	
80–89	36	3.2	7.0	22.2		16.7		19.4		0.0		8.3		11.1		19.4	
≥90	3	0.3	10.0	33.3		0.0		33.3		0.0		33.3		0.0		33.3	
Gender					0.120		0.288		0.499		0.125		0.105		0.276		0.772
Female	593	51.9	8.0	21.1		7.6		11.6		0.7		12.5		10.5		4.6	
Male	549	48.1	9.0	25.0		9.5		10.4		1.6		9.5		8.6		4.9	
Cohort					<0.001		0.011		<0.001		0.224		0.066		0.010		<0.001
Endovascular neurosurgery/ Cerebrovascular neurosurgery	284	24.9	8.0	31.3		5.6		17.6		1.1		16.2		8.8		9.5	
Oncological neurosurgery	356	31.2	9.0	18.0		9.6		5.6		0.8		8.1		9.0		5.9	
Epilepsy surgery	36	3.2	9.0	13.9		0.0		8.3		2.8		8.3		5.6		0.0	
Pituitary surgery	52	4.6	8.5	17.3		13.5		5.8		1.9		15.4		23.1		0.0	
Hydrocephalus	25	2.2	14.0	52.0		16.0		36.0		0.0		4.0		28.0		8.0	
Functional neurosurgery	31	2.7	9.0	25.8		6.5		9.7		0.0		12.9		6.5		0.0	
Spinal neurosurgery	177	15.5	6.0	7.3		5.6		5.6		0.0		8.5		6.8		0.0	
Pediatric neurosurgery	162	14.2	10.0	35.2		13.0		16.0		3.1		11.7		9.3		2.5	
Miscellaneous	19	1.7	8.0	21.1		15.8		10.5		0.0		5.3		10.5		0.0	
BMI WHO categories					0.035		0.429		0.001		0.359		0.030		0.884		0.025
<18.5	71	6.2	10.0	28.2		15.5		16.9		1.4		11.3		7.0		1.4	
18.5–24.9	301	26.4	8.0	17.9		8.6		8.0		0.3		9.6		9.6		2.7	
25–29.9	228	20.0	8.0	19.3		7.0		10.1		1.8		8.8		8.3		3.1	
30–34.9	115	10.1	8.0	22.6		7.0		3.5		1.7		5.2		10.4		5.2	
35–39.9	35	3.1	9.0	25.7		5.7		11.4		2.9		17.1		11.4		8.6	
≥40	13	1.1	8.0	15.4		0.0		0.0		0.0		23.1		0.0		7.7	
None assigned	379	33.2	9.0	28.2		9.0		15.6		1.1		14.2		10.6		7.4	
ASA classification					<0.001		0.058		<0.001		0.240		<0.001		0.172		<0.001
I	212	18.6	8.0	9.9		6.6		5.2		0.0		8.0		8.0		0.5	
II	525	46.0	8.0	17.5		9.1		8.0		1.1		9.1		9.0		2.3	
III	229	20.1	9.0	31.4		8.7		11.8		2.2		10.9		10.0		6.6	
IV	45	3.9	13.0	46.7		2.2		28.9		2.2		22.2		4.4		15.6	
V	30	2.6	15.0	53.3		23.3		30.0		0.0		30.0		10.0		33.3	
None assigned	101	8.8	11.0	39.6		6.9		23.8		1.0		16.8		16.8		8.9	
Presence of a chronic comorbidity	769	67.3	8.0	23.5	0.709	8.7	0.897	10.5	0.575	0.9	0.442		0.215	10.1	0.243	5.9	0.038
Charlson Comorbidity Index					0.010		0.340		0.752		0.679		0.737		0.611		<0.001
0	667	58.4	8.0	19.6		9.0		11.5		1.2		10.0		10.0		3.3	
1	146	12.8	8.0	23.3		4.1		8.9		1.4		13.0		6.8		5.5	
2	100	8.8	9.0	32.0		10.0		11.0		0.0		13.0		7.0		10.0	
≥3	76	6.7	9.5	31.6		10.5		7.9		0.0		13.2		10.5		14.5	
Unknown	153	13.4	9.0	26.8		8.5		12.4		2.0		11.1		11.1		2.0	
Comorbidities																	
Myocardial infarction	25	2.2	8.0	24.0	0.894	12.0	0.542	4.0	0.289	0.0	0.998	12.0	0.886	12.0	0.622	8.0	0.495
Congestive heart failure	4	0.4	5.0	25.0	0.920	25.0	0.272	0.0	0.999	0.0	0.999	50.0	0.037	0.0	0.999	50.0	0.003
Coronary heart disease	36	3.2	8.0	22.2	0.913	5.6	0.512	11.1	0.953	0.0	0.998	25.0	0.009	8.3	0.844	5.6	0.881
Cardiac arrhythmias	70	6.1	7.0	22.9	0.981	5.7	0.378	11.4	0.863	1.4	0.758	15.7	0.218	8.6	0.836	11.4	0.014
Valvular disease	20	1.8	7.0	30.0	0.448	5.0	0.568	15.0	0.546	0.0	0.999	10.0	0.873	5.0	0.521	15.0	0.053
Peripheral vascular disease	27	2.4	8.0	33.3	0.196	3.7	0.374	11.1	0.961	0.0	0.998	3.7	0.242	7.4	0.747	11.1	0.155
Prior pulmonary embolism	15	1.3	9.0	20.0	0.787	20.0	0.128	6.7	0.605	0.0	0.999	13.3	0.784	6.7	0.735	13.3	0.158
Prior deep vein thrombosis	14	1.2	11.0	28.6	0.614	28.6	0.014	7.1	0.658	0.0	0.999	7.1	0.637	7.1	0.790	7.1	0.717
Chronic pulmonary disease	76	6.7	8.5	26.3	0.462	3.9	0.145	9.2	0.639	1.3	0.824	7.9	0.357	10.5	0.673	10.5	0.027
Hypertension	315	27.6	9.0	26.3	0.079	6.3	0.091	9.8	0.507	1.6	0.284	12.7	0.280	6.0	0.022	7.9	0.005
Dyslipidemia	123	10.8	8.0	23.6	0.849	8.1	0.843	13.8	0.256	2.4	0.129	13.8	0.310	9.8	0.813	8.1	0.098
Diabetes without end-organ damage	63	5.5	10.0	34.9	0.021	9.5	0.787	6.3	0.245	0.0	0.997	11.1	1.000	6.3	0.425	6.3	0.620
Diabetes with end-organ damage	3	0.3	24.0	66.7	0.119	0.0	0.999	33.3	0.247	0.0	0.999	33.3	0.257	66.7	0.015	0.0	0.999
Cerebrovascular disease	64	5.6	7.5	25.0	0.676	7.8	0.819	15.6	0.204	1.6	0.698	10.9	0.966	7.8	0.698	7.8	0.296
Hemiplegia or paraplegia	15	1.3	14.0	53.3	0.003	13.3	0.484	20.0	0.160	0.0	0.999	13.3	0.721	13.3	0.549	20.0	0.004
Hypothyroidism	80	7	9.0	16.3	0.137	10.0	0.660	8.8	0.524	1.3	0.863	13.8	0.448	8.8	0.849	7.5	0.292
Hyperthyroidism	3	0.3	12.0	33.3	0.675	0.0	0.999	0.0	0.999	0.0	0.999	0.0	0.999	33.3	0.197	0.0	0.999
Mild liver disease	22	1.9	12.0	40.9	0.051	13.6	0.409	0.0	0.998	0.0	0.998	13.6	0.713	4.5	0.445	13.6	0.075
Moderate/severe liver disease	2	0.2	26.0	50.0	0.393	0.0	0.999	0.0	0.999	0.0	1.000	50.0	0.142	0.0	0.999	0.0	0.999
Ulcer disease	6	0.5	8.5	0.0	0.999	0.0	0.999	0.0	0.999	0.0	0.999	16.7	0.667	0.0	0.999	0.0	0.999
Coagulopathy	14	1.2	8.0	21.4	0.895	14.3	0.451	21.4	0.211	0.0	0.999	14.3	0.705	28.6	0.019	7.1	0.716
Blood loss anemia	0	0	N/A	0.0	N/A	0.0	N/A	0.0	N/A	0.0	N/A	0.0	N/A	0.0	N/A	0.0	N/A
Deficiency anemia	6	0.5	8.5	16.7	0.717	16.7	0.490	0.0	0.999	0.0	0.999	0.0	0.999	0.0	0.999	0.0	0.999
Connective tissue disease	13	1.1	6.0	7.7	0.219	15.4	0.389	15.4	0.597	0.0	0.999	15.4	0.624	7.7	0.852	7.7	0.660
Fluid and electrolyte disorders	8	0.7	8.5	37.5	0.334	12.5	0.695	25.0	0.214	0.0	0.999	12.5	0.900	12.5	0.745	12.5	0.351
Moderate/severe renal disease	17	1.5	10.0	35.3	0.234	5.9	0.682	5.9	0.512	0.0	0.999	11.8	0.939	5.9	0.624	23.5	0.002
HIV	4	0.4	12.0	50.0	0.225	0.0	0.999	0.0	0.999	0.0	0.999	25.0	0.394	25.0	0.305	0.0	0.999
AIDS	0	0	N/A	0.0	N/A	0.0	N/A	0.0	N/A	0.0	N/A	0.0	N/A	0.0	N/A	0.0	N/A
Any tumor	95	8.3	9.0	30.5	0.065	11.6	0.279	13.7	0.347	0.0	0.997	14.7	0.240	8.4	0.788	11.6	0.003
Leukemia	2	0.2	11.0	50.0	0.390	0.0	0.999	0.0	0.999	0.0	1.000	50.0	0.141	0.0	0.999	0.0	0.999
Lymphoma	5	0.4	14.0	60.0	0.075	0.0	0.999	0.0	0.999	0.0	0.999	0.0	0.999	0.0	0.999	0.0	0.999
Metastatic solid tumor	24	2.1	10.0	33.3	0.223	20.8	0.039	0.0	0.998	0.0	0.998	16.7	0.385	8.3	0.885	20.8	0.001
Depression	34	3	11.5	41.2	0.012	11.8	0.505	20.6	0.069	2.9	0.301	8.8	0.667	17.6	0.090	5.9	0.816
Dementia	7	0.6	7.0	0.0	0.999	0.0	0.999	14.3	0.773	0.0	0.999	0.0	0.999	14.3	0.655	14.3	0.285
Psychosis	4	0.4	10.5	25.0	0.920	50.0	0.018	0.0	0.999	0.0	0.999	25.0	0.395	0.0	0.999	0.0	0.999
Alcohol abuse	19	1.7	6.0	10.5	0.213	10.5	0.761	5.3	0.444	0.0	0.999	21.1	0.173	0.0	0.998	15.8	0.042
Drug abuse	8	0.7	8.0	0.0	0.999	0.0	0.999	0.0	0.999	0.0	0.999	12.5	0.899	0.0	0.999	0.0	0.999
Nicotine abuse	71	6.2	9.0	28.2	0.280	8.5	0.967	12.7	0.598	0.0	0.997	15.5	0.224	9.9	0.856	7.0	0.420
Total number of preoperative medications					0.001		0.586		0.046		0.344		0.279		0.677		0.527
0	202	17.7	8.0	17.8		7.9		11.9		0.5		9.4		10.9		4.0	
1	156	13.7	8.0	16.7		7.1		9.6		0.6		11.5		7.1		3.2	
2	131	11.5	8.0	18.3		6.1		6.9		2.3		6.1		8.4		3.1	
≥3	416	36.4	9.0	24.5		8.9		9.6		1.7		12.5		9.4		5.8	
Unknown	237	20.8	9.0	31.2		10.5		16.0		0.4		12.2		11.0		5.5	
Preoperative medications																	
Acetylsalicylic acid	104	9.1	8.0	21.2	0.943	4.8	0.221	11.5	0.485	1.0	0.748	13.5	0.338	7.7	0.599	6.7	0.260
Antiplatelet (other than acetylsalicylic acid)	15	1.3	7.0	26.7	0.602	13.3	0.450	6.7	0.685	6.7	0.105	0.0	0.999	0.0	0.999	0.0	0.999
Anticoagulant	98	8.6	10.0	33.7	0.002	9.2	0.636	13.3	0.227	1.0	0.791	14.3	0.258	13.3	0.152	14.3	<0.001
NSAID (other than acetylsalicylic acid)	55	4.8	7.0	10.9	0.059	9.1	0.760	9.1	0.863	0.0	0.998	9.1	0.665	12.7	0.373	1.8	0.329
Steroid	133	11.6	9.0	27.8	0.044	18.0	<0.001	7.5	0.346	0.8	0.543	9.8	0.692	12.8	0.127	8.3	0.033
Antiepileptic drug	212	18.6	9.0	23.6	0.273	7.5	0.839	7.1	0.153	2.4	0.139	11.3	0.794	7.1	0.226	5.2	0.638
Opioid	62	5.4	7.0	19.4	0.747	6.5	0.661	11.3	0.653	0.0	0.997	14.5	0.325	9.7	0.898	11.3	0.013
Antidepressant	121	10.6	8.0	25.6	0.170	9.9	0.382	9.9	0.921	2.5	0.241	6.6	0.118	9.1	0.954	5.8	0.468
Angiotensin-converting-enzyme inhibitor	84	7.4	8.0	17.9	0.484	3.6	0.126	4.8	0.118	2.4	0.381	15.5	0.128	3.6	0.072	8.3	0.083
Angiotensin II receptor blocker	136	11.9	8.5	28.7	0.015	8.8	0.716	10.3	0.797	0.7	0.524	15.4	0.047	8.8	0.852	5.1	0.701
Diuretic	91	8	8.0	26.4	0.170	8.8	0.781	11.0	0.659	2.2	0.447	15.4	0.119	7.7	0.589	11.0	0.003
Calcium channel blocker	125	10.9	9.0	28.0	0.034	9.6	0.490	12.8	0.208	0.0	0.996	14.4	0.136	11.2	0.419	7.2	0.125
Insulin	17	1.5	11.0	41.2	0.048	11.8	0.567	5.9	0.591	0.0	0.999	17.6	0.360	11.8	0.714	5.9	0.800
Hypoglycemic oral drug	53	4.6	9.0	37.7	0.003	7.5	0.901	7.5	0.579	0.0	0.998	15.1	0.294	11.3	0.583	3.8	0.768
Statin	141	12.3	8.0	25.5	0.159	9.2	0.566	12.1	0.321	2.1	0.366	13.5	0.259	9.2	1.000	6.4	0.278
β-blocker	146	12.8	8.0	28.1	0.019	8.2	0.931	9.6	0.964	1.4	0.956	14.4	0.104	7.5	0.436	6.8	0.143
Immunosuppressant	13	1.1	8.0	23.1	0.859	0.0	0.999	7.7	0.800	0.0	0.999	7.7	0.721	7.7	0.848	0.0	0.999

The evaluated operative metrics and their outcomes are summarized in Table 2. The preoperative laboratory values of the patients and their outcomes are shown in Table 1 in the S1 Appendix. The assessments of additional patient characteristics and clinical parameters are reported in Table 2 in the S1 Appendix. A subgroup analysis of pediatric patients in various age groups is shown in Table 3 in the S1 Appendix.

**Table 2:** Operative parameters and outcomes.

	No. of patients Total = 1142		LOS at Index Admission (in days)	Proportion of Patients with an Extended LOS		90-Day Unplanned Readmission Rate		90-Day Unplanned Reoperation Rate		Preoperative Event Rate		Intraoperative Event Rate		Postoperative Event Rate		90-Day Postoperative Mortality Rate	
Variables	N	%	Median	%	*p*-value	%	*p*-value	%	*p*-value	%	*p*-value	%	*p*-value	%	*p*- value	%	*P-* value
Admission status					<0.001		0.002		<0.001		0.279		0.319		0.022		<0.001
Home	764	66.9	8.0	11.9		6.7		5.9		1.0		9.7		8.1		0.9	
Transfer from another department	50	4.4	17.5	62.0		20.0		16.0		2.0		12.0		12.0		18.0	
Transfer from another hospital	211	18.5	11.0	41.7		10.9		23.7		0.5		13.7		10.0		10.4	
ER/Trauma surgery/Shock room	107	9.4	12.0	42.1		9.3		15.9		2.8		15.0		15.9		13.1	
Birth admission	9	0.8	47.0	77.8		33.3		66.7		0.0		11.1		33.3		22.2	
Unknown	1	0.1	3.0	0.0		0.0		0.0.		0.0		0.0		0.0		0.0	
Day of index operation					<0.001		0.695		<0.001		0.616		0.637		0.276		0.077
Weekday	1048	91.8	8.0	21.0		8.4		9.8		1.2		11.2		9.8		4.4	
Weekend	94	8.2	10.5	44.7		9.6		24.5		0.0		9.6		6.4		8.5	
Operation length in groups (minutes)					0.149		0.124		<0.001		0.611		0.197		0.016		0.024
<100	513	44.9	8.0	25.3		10.3		15.0		1.2		9.4		9.6		6.8	
100–199	503	44.0	9.0	19.9		6.4		7.0		1.0		13.3		7.8		3.4	
200–299	84	7.4	9.0	27.4		9.5		10.7		2.4		9.5		14.3		1.2	
≥300	42	3.7	10.0	21.4		9.5		11.9		0.0		7.1		21.4		2.4	
Total operating room time in groups (minutes)					<0.001		0.145		<0.001		0.301		0.840		<0.001		<0.001
<100	114	10.0	12.5	47.4		14.0		34.2		1.8		10.5		18.4		15.8	
100–199	580	50.8	7.0	19.1		7.4		7.9		0.7		10.5		5.7		4.3	
200–299	323	28.3	9.0	19.8		8.4		9.0		1.9		12.4		10.5		2.5	
≥300	125	10.9	10.0	26.4		8.8		9.6		0.8		10.4		16.8		2.4	
Type of anesthesia					0.230		0.244		0.545		0.747		0.215		0.208		0.450
General anesthesia/Sedoanalgesia	1028	90.0	9.0	23.0		8.8		11.1		1.2		11.1		9.9		4.9	
General anesthesia with awake phase	1	0.1	14.0	100		0.0		0.0		0.0		0.0		0.0		0.0	
Local anesthesia	40	3.5	2.0	15.0		0.0		5.0		0.0		20.0		0.0		0.0	
Standby anesthesia	13	1.1	4.0	15.4		7.7		7.7		0.0		0.0		7.7		7.7	
Unknown	60	5.3	9.0	28.3		10.0		15.0		1.7		6.7		10.0		5.0	
Start time of index operation					<0.001		0.380		<0.001		0.193		0.276		0.019		<0.001
7:00–14:59	916	80.2	8.0	18.2		8.0		7.2		1.3		10.4		8.4		2.9	
15:00–22:59	181	15.8	10.0	39.2		10.5		25.4		0.0		14.4		13.3		12.7	
23:00–6:59	45	3.9	16.0	53.3		11.1		31.1		2.2		11.1		17.8		8.9	
Surgical urgency					<0.001		0.425		<0.001		0.349		0.002		0.034		<0.001
Elective	789	69.1	8.0	15.5		8.0		5.7		1.0		9.3		8.1		2.3	
Urgent	161	14.1	8.0	34.2		9.9		18.0		2.5		10.6		11.8		6.2	
Emergency (within 2 hours)	89	7.8	14.0	50.6		12.4		31.5		0.0		21.3		10.1		19.1	
None assigned	103	9.0	10.0	38.8		6.8		23.3		1.0		16.5		16.5		8.7	
Surgical positioning					<0.001		0.209		<0.001		0.669		0.089		0.016		<0.001
Supine position	719	63.0	9.0	25.5		9.3		11.1		1.7		11.0		9.5		5.0	
Prone position	177	15.5	8.0	16.4		6.8		7.3		0.6		7.9		5.6		4.0	
Sitting position	75	6.6	9.0	14.7		2.7		8.0		0.0		13.3		12.0		1.3	
Lateral position	14	1.2	9.0	21.4		14.3		28.6		0.0		28.6		21.4		0.0	
Trendelenburg position	3	0.3	94.0	100		33.3		66.7		0.0		0.0		66.7		0.0	
Reverse Trendelenburg position	2	0.2	5.5	0.0		0.0		0.0		0.0		0.0		0.0		0.0	
Concorde position	1	0.1	10.0	0.0		0.0		0.0		0.0		0.0		0.0		0.0	
Discus position	100	8.8	6.0	7.0		7.0		6.0		0.0		8.0		8.0		0.0	
Unknown	51	4.5	14.0	51.0		11.8		29.4		0.0		21.6		17.6		19.6	
Number of neurosurgical operations during index hospital stay in categories					<0.001		0.373		<0.001		0.519		0.001		<0.001		<0.001
1	987	86.4	8.0	15.5		8.1		3.7		1.1		9.8		6.1		3.9	
2	101	8.8	16.0	62.4		10.9		44.6		2.0		15.8		22.8		7.9	
3+	54	4.7	34.5	85.2		11.1		81.5		0.0		24.1		48.1		14.8	
Total length of hospital stay at index admission in categories (in days)					N/A		0.003		<0.001		0.084		<0.001		<0.001		<0.001
1–7	478	41.9	N/A	N/A		5.6		4.6		0.8		9.2		3.3		3.6	
8–14	437	38.3	N/A	N/A		8.7		6.9		0.7		8.7		7.8		2.7	
15–21	96	8.4	N/A	N/A		13.5		16.7		3.1		18.8		19.8		14.6	
>21	131	11.5	N/A	N/A		14.5		44.3		2.3		19.8		30.5		8.4	
ICU stay during index hospital admission					<0.001		0.868		<0.001		0.779		0.183		0.008		<0.001
Yes	727	63.7	9.0	28.7		8.4		13.6		1.2		12.0		11.3		6.3	
No	415	36.3	7.0	12.8		8.7		6.5		1.0		9.4		6.5		1.9	
Discharge disposition after surgical treatment					<0.001		0.033		<0.001		0.657		0.022		0.004		<0.001
Home	882	77.2	8.0	15.3		9.1		7.3		1.0		10.1		7.8		1.0	
Transfer to another department	59	5.2	23.0	78.0		6.8		11.9		1.7		13.6		16.9		8.5	
Transfer to another hospital	155	13.6	11.0	38.1		5.2		25.8		1.9		12.3		15.5		8.4	
Nursing home	4	0.4	67.0	100		25.0		25.0		0.0		25.0		25.0		0.0	
Rehabilitation center	2	0.2	44.5	50.0		0.0		50.0		0.0		50.0		50.0		0.0	
Hospice	2	0.2	19.0	100		0.0		0.0		0.0		0.0		0.0		100.0	
Patient died	26	2.3	13.0	46.2		0.0		38.5		0.0		30.8		15.4		92.3	
Unknown	12	1.1	8.5	25.0		33.3		25.0		0.0		0.0		0.0		8.3	
Discharge against medical advice					0.019		0.136		<0.001		1.000		0.012		0.231		<0.001
Yes	11	1.0	9.0	18.2		18.2		27.3		0.0		9.1		18.2		0.0	
No	1105	96.8	8.0	22.4		8.6		10.2		1.2		10.6		9.3		2.7	
Patient died	26	2.3	13.0	46.2		0.0		38.5		0.0		30.8		15.4		92.3	

In the study cohort, the most common diagnoses at the index hospital admission were primary brain tumor (n = 334, 29.2%), unruptured cerebral aneurysm (n = 75, 6.6%), secondary brain tumor (n = 70, 6.1%), epilepsy (n = 68, 6%), and subdural hemorrhage (n = 61, 5.3%) (Table 3). Among the 368 patients treated for a primary cranial or spinal tumor, meningioma (n = 97, 26.4%), glioblastoma (n = 51, 13.9%), vestibular schwannoma (n = 48, 13%), pituitary adenoma (n = 42, 11.4%), and pilocytic astrocytoma (n = 13, 3.5%) were the most frequent tumor histologies (Table 4 in the S1 Appendix). WHO grade I (n = 142, 38.6%) and WHO grade IV (n = 58, 15.8%) tumors represented most of the primary neoplasms in the patient cohort (Table 5 in the S1 Appendix). The main origins of the secondary tumors (n = 72) were lung cancer (n = 38, 47.2%), breast cancer (n = 8, 11.1%), B-cell lymphoma (n = 5, 6.9%), and melanoma (n = 4, 5.6%) (Table 6 in the S1 Appendix).

**Table 3:** Primary diagnoses at the index hospital admission and outcomes.

	No. of patients Total = 1142		LOS at Index Admission (in days)	Proportion of Patients with an Extended LOS	90-Day Unplanned Readmission Rate	90-Day Unplanned Reoperation Rate	Preoperative Event Rate	Intraoperative Event Rate	Postoperative Event Rate	90-Day Postoperative Mortality Rate
Primary Diagnosis	N	%	Median	%	%	%	%	%	%	%
Unruptured cerebral aneurysm	75	6.6	6.0	2.7	1.3	2.7	0.0	20.0	2.7	1.3
Ruptured cerebral aneurysm with subarachnoid hemorrhage	60	5.3	17.0	65.0	6.7	38.3	0.0	20.0	30.0	15.0
Non-aneurysmal subarachnoid hemorrhage	13	1.1	9.0	38.5	7.7	23.1	0.0	23.1	0.0	0.0
Intracerebral hemorrhage	40	3.5	13.5	50.0	2.5	15.0	0.0	15.0	2.5	27.5
Subdural hemorrhage	61	5.3	7.0	21.3	14.8	18.0	0.0	9.8	1.6	13.1
Epidural hemorrhage	1	0.1	10.0	0.0	0.0	0.0	0.0	100.0	0.0	0.0
Intraventricular hemorrhage with hydrocephalus	6	0.5	79.5	100.0	33.3	50.0	0.0	0.0	33.2	0.0
Arteriovenous malformation	15	1.3	6.0	26.7	13.3	6.7	6.7	0.0	0.0	0.0
Arteriovenous fistula	15	1.3	6.0	13.3	0.0	0.0	6.7	6.7	0.0	0.0
Moyamoya disease	2	0.2	4.5	0.0	0.0	0.0	0.0	50.0	0.0	0.0
Cerebral venous sinus thrombosis	1	0.1	2.0	0.0	0.0	100.0	0.0	0.0	0.0	100.0
Hemangioma	17	1.5	7.0	5.9	5.9	5.9	0.0	5.9	5.9	0.0
Primary brain tumor	334	29.2	9.0	19.8	9.9	7.2	0.6	10.2	12.6	2.4
Secondary brain tumor	70	6.1	10.0	25.7	21.4	8.6	1.4	7.1	8.6	18.6
Tumor on the skull and facial bones	7	0.6	5.0	0.0	14.3	0.0	0.0	14.3	0.0	0.0
Cerebral cyst	17	1.5	7.0	11.8	11.8	11.8	0.0	5.9	11.8	0.0
Intracranial abscess	4	0.4	24.5	75.0	25.0	50.0	0.0	0.0	0.0	0.0
Other cerebral lesion	22	1.9	5.5	18.2	4.5	4.5	4.5	9.1	0.0	0.0
Epilepsy	68	6.0	11.0	32.4	1.5	7.4	5.9	10.3	5.9	0.0
Hydrocephalus	35	3.1	14.0	54.3	20.0	40.0	0.0	8.6	25.7	8.6
Cerebral infection	2	0.2	16.0	50.0	50.0	0.0	0.0	0.0	0.0	0.0
Trigeminal neuralgia	15	1.3	6.0	0.0	13.3	20.0	0.0	20.0	13.3	0.0
Parkinson’s disease	8	0.7	12.5	37.5	0.0	0.0	0.0	0.0	0.0	0.0
Depression	6	0.5	15.5	83.3	0.0	0.0	0.0	0.0	0.0	0.0
Arnold-Chiari malformation	6	0.5	7.0	0.0	0.0	0.0	0.0	0.0	0.0	0.0
Tethered cord syndrome	6	0.5	14.0	50.0	0.0	16.7	0.0	0.0	16.7	0.0
Spina bifida	1	0.1	9.0	0.0	100.0	0.0	0.0	0.0	0.0	0.0
Craniosynostosis	23	2.0	8.0	8.7	4.3	4.3	0.0	17.4	4.3	0.0
Disc herniation (Cervical)	23	2.0	6.0	0.0	0.0	4.3	0.0	13.0	4.3	0.0
Disc herniation (Cervicothoracic)	2	0.2	8.0	0.0	50.0	50.0	0.0	50.0	50.0	0.0
Disc herniation (Thoracic)	3	0.3	5.0	0.0	33.3	0.0	0.0	0.0	0.0	0.0
Disc herniation (Lumbar)	39	3.4	6.0	5.1	10.3	10.3	0.0	7.7	10.3	0.0
Disc herniation (Lumbosacral)	29	2.5	6.0	0.0	3.4	3.4	0.0	10.3	3.4	0.0
Spondylosis	4	0.4	6.0	0.0	0.0	0.0	0.0	0.0	0.0	0.0
Spinal stenosis	32	2.8	6.5	18.8	0.0	3.1	0.0	9.4	6.3	0.0
Primary spinal tumor	27	2.4	7.0	3.7	3.7	3.7	0.0	3.7	3.7	0.0
Secondary spinal tumor	2	0.2	20.0	50.0	0.0	0.0	0.0	0.0	0.0	0.0
Spinal cyst	7	0.6	6.0	14.3	0.0	0.0	0.0	14.3	0.0	0.0
Spinal abscess	1	0.1	9.0	0.0	0.0	100.0	0.0	0.0	100.0	0.0
Other spinal lesion	6	0.5	8.0	16.7	16.7	0.0	0.0	0.0	16.7	0.0
Miscellaneous	37	3.2	9.0	27.0	2.7	16.2	8.1	13.5	13.5	0.0

The most frequently performed procedures at the index operation were removal of an intracerebral mass in the cerebral hemispheres (n = 174, 15.2%), catheter angiography of the head and neck (n = 77, 6.7%), removal of extracerebral tumors at the skull base of the brain (n = 63, 5.5%), decompression of lumbar nerve roots (n = 63, 5.5%), transsphenoidal resection of an adenoma (n = 51, 4.5%), placement of an external ventricular drain through burr hole trepanation (n = 46, 4%), evacuation of a subdural hematoma through burr hole trepanation (n = 45, 3.9%), removal of a cerebellar tumor (n = 41, 3.6%), resection of a vestibular schwannoma (n = 40, 3.5%), and resection of epileptogenic foci (n = 31, 2.7%). The outcomes for the most frequent index surgeries are summarized in Table 4.

**Table 4:** Patient outcomes for the most frequently performed index procedures.

	No. of patients Total = 1142		LOS at Index Admission (in days)	Proportion of Patients with an Extended LOS	90-Day Unplanned Readmission Rate	90-Day Unplanned Reoperation Rate	Preoperative Event Rate	Intraoperative Event Rate	Postoperative Event Rate	90-Day Postoperative Mortality Rate
Index Procedure	N	%	Median	%	%	%	%	%	%	%
Removal of an intracerebral mass in the cerebral hemispheres	174	15.2	10.0	24.1	12.1	6.3	0.6	6.9	8.0	6.3
Catheter angiography of the head and neck	77	6.7	3.0	16.9	5.2	5.2	1.3	13.0	2.6	0.0
Removal of extracerebral tumors at the base of the brain	63	5.5	9.0	11.1	6.3	6.3	1.6	6.3	15.9	0.0
Decompression of lumbar nerve roots	63	5.5	6.0	3.2	7.9	7.9	0.0	6.3	9.5	0.0
Transsphenoidal resection of an adenoma	51	4.5	8.0	17.6	13.7	5.9	2.0	15.7	23.5	0.0
Placement of an external ventricular drain by burr hole trepanation	46	4.0	25.5	80.4	10.9	54.3	0.0	13.0	41.3	19.6
Evacuation of a subdural hematoma by burr hole trepanation	45	3.9	7.0	22.2	17.8	22.2	0.0	4.4	2.2	17.8
Removal of a cerebellar tumor	41	3.6	9.0	17.1	4.9	9.8	0.0	14.6	9.8	2.4
Resection of a vestibular schwannoma	40	3.5	9.0	5.0	2.5	0.0	0.0	5.0	7.5	0.0
Resection of epileptogenic foci	31	2.7	10.0	12.9	3.2	6.5	3.2	6.5	3.2	0.0

### Length of hospital stay

The overall LOS of the entire patient cohort at the index hospitalization ranged from a 1 to 242 days. On average, the patients spent a median of 8 days at the hospital during the index admission. The median LOS until the index operation was 1 day (range: 0–50 days), whereas the median LOS after the index operation was 7 days (range: 0–242 days). Patients with an extended LOS remained in the hospital for a median of 21.5 days (range: 14–242 days). In contrast, patients without prolonged hospitalization stayed in the hospital for a median of 7 days (range: 1–13 days). The cohort of patients with hydrocephalus had the longest median LOS (median 14 days, range 3–111 days), whereas the shortest median LOS was observed among patients undergoing spinal neurosurgery (median 6 days, range 1–74 days) (Table 1). The median length of the index hospitalization for the most frequent tumor histologies was 9 days for meningioma (range: 3–127 days), 13 days for glioblastoma (range: 2–87 days), 9 days for vestibular schwannoma (5–53 days), 8 days for pituitary adenoma (range: 5–30 days), and 10 days for pilocytic astrocytoma (range: 6–21 days). Patients with WHO grade I, II, or III tumors spent a median of 9 days in the hospital during the index stay, whereas patients with WHO grade IV tumors remained in the hospital for a median of 13 days.

### Extended length of hospital stay

Of the 1142 patients in the study cohort, 262 (22.9%) had an extended LOS, i.e., hospitalization for ≥14 days. The prevalence of extended LOS was highest among neonates (83.3%, n = 10), followed by toddlers 12 months to <2 years of age (37.5%, n = 3), and young children 2 to <6 years of age (36.7%, n = 11).

Patient characteristics predictive of an extended LOS in univariate analyses were high Charlson Comorbidity Index score (OR 1.89, *p* = 0.010); presence of hemiplegia or paraplegia (OR 4.69, *p* = 0.003), depression (OR 2.44, *p* = 0.012), or diabetes without end-organ damage (OR 1.89, *p* = 0.021); high number of preoperative medications (OR 2.09, *p* = 0.001); preoperative use of anticoagulants (OR 2.04, *p* = 0.002), steroids (OR 1.54, *p* = 0.044), angiotensin II receptor blockers (OR 1.67, *p* = 0.015), calcium channel blockers (OR 1.59, *p* = 0.034), insulin (OR 2.69, *p* = 0.048), hypoglycemic oral drugs (OR 2.42, *p* = 0.003), or β-blockers (OR 1.62, *p* = 0.019); and high ASA grade (OR 10.40, *p*<0.001) An assessment of social parameters indicated that patients who were unable to independently perform daily activities in the home environment were relatively more likely to have an extended LOS at the index hospitalization (OR 0.24, *p*<0.001).

Multivariate analysis indicated that being in the cohort of patients with hydrocephalus (OR 7.69, 95% CI 1.75–33.85, *p* = 0.007), preoperative albumin of <35 g/L (OR 4.05, 95% CI 1.38–11.92, *p* = 0.011), preoperative prothrombin time >125% (Owren) (OR 2.53, 95% CI 1.04–6.17, *p* = 0.041), presence of preoperative depression (OR 3.34, 95% CI 1.04–10.69, *p =* 0.043), transfer from another department (OR 15.79, 95% CI 5.52–45.17, *p*<0.001), transfer from another hospital (OR 2.76, 95% CI 1.36–5.61, *p* = 0.005), two neurosurgical operations during the index hospital stay (OR 5.66, 95% CI 2.88–11.11, *p*<0.001), three or more neurosurgical operations during the index hospital stay (OR 11.07, 95% CI 3.39–36.13, *p*<0.001), intensive care unit stay (OR 2.41, 95% CI 1.30–4.49, *p* = 0.005), readmission to the intensive care unit during the index hospital stay (OR 5.14, 95% CI 1.07–24.01, *p* = 0.041), intraoperative event at the index operation (OR 2.77, 95% CI 1.47–5.23, *p* = 0.002), postoperative event within 90 days after the index operation (OR 5.52, 95% CI 2.76–11.04, *p*<0.001), and transfer to another department after surgical treatment (OR 54.02, 95% CI 16.71–174.66, *p*<0.001) were independently associated with greater likelihood of an extended hospital stay.

### Readmissions

Among the 1142 participants, the all-cause 7-, 15-, 30-, 60-, and 90-day readmission rates were 3.9% (n = 44), 5.7% (n = 65), 8.8% (n = 100), 12.3% (n = 141), and 16.5% (n = 188), respectively. The median duration until readmission was 34 days (range: 0–90 days) after discharge from the index hospitalization and 48 days (range: 4–196 days) after the index operation (Fig 1). Of the 247 readmissions, 127 (51.4%) were planned, and 120 (48.6%) were unplanned. The 7-, 15-, 30-, 60-, and 90-day unplanned readmission rates were 2.8% (n = 32), 3.7% (n = 42), 5.3% (n = 61), 7.0% (n = 80), and 8.5% (n = 97), respectively. Patients underwent neurosurgical operation in approximately one-third of readmissions (n = 82, 33.2%). More than three-quarters of readmissions (n = 188, 76.1%) were first-time rehospitalizations; the maximum number of readmission episodes within 90 days after discharge from the index hospital admission was 7 (n = 1, 0.4% of readmission). The median LOS was 4 days (range: 0–56 days) for planned readmission and 5.5 days (range: 0–94 days) for unplanned readmission.

Unplanned readmission was due primarily to medical and/or neurological deterioration (n = 26, 21.67%), wound complications (n = 12, 10%), and non-central nervous system infection (n = 11, 9.17%) (Table 5).

**Table 5:** Causes of unplanned readmission.

Causes of Unplanned Readmissions		N	%
Deterioration of the medical and/or neurological situation		26	21.67
Wound complications		12	10.00
Non-central nervous system infection		11	9.17
Seizures		7	5.83
Shunt-associated complications		6	5.00
Traumatic injury		5	4.17
Aplasia		5	4.17
Hydrocephalus		5	4.17
Recurrence of chronic subdural hematoma		4	3.33
Fever with/without cytopenia		4	3.33
Subcutaneous cerebrospinal fluid accumulation		4	3.33
Pain		4	3.33
Hyponatremia		4	3.33
Central nervous system infection		3	2.50
Gastrointestinal complications		3	2.50
Epistaxis		2	1.67
Recurrence of spinal disc herniation		2	1.67
Syndrome of inappropriate secretion of antidiuretic hormone		1	0.83
Diagnosis of a new metastatic lesion		1	0.83
Thrombocytopenia		1	0.83
Rhinoliquorrhea		1	0.83
Vertigo and nausea		1	0.83
Acute respiratory insufficiency		1	0.83
Cerebrospinal fluid leakage		1	0.83
Infection with an unknown focus		1	0.83
Miscellaneous		3	2.50

The most frequently documented diagnoses at unplanned readmission were “C71.9 Malignant neoplasm: Brain, unspecified” (n = 13, 10.83%), “T81.3 Disruption of operation wound, not elsewhere classified” (n = 11, 9.17%), and “C79.3 Secondary malignant neoplasm of brain and cerebral meninges” (n = 5, 4.17%).

According to the logistic regression model, a preoperative sodium level of <135 mmol/L (OR 4.35, 95% CI 1.48–12.80, *p* = 0.008), prior deep vein thrombosis (OR 5.05, 95% CI 1.17–21.87, *p* = 0.030), presence of psychosis preoperatively (OR 16.74, 95% CI 1.82–153.90, *p* = 0.013), being in the pediatric neurosurgery cohort (OR 2.49, 95% CI 1.01–6.12, *p* = 0.047), transfer from another department during the index hospitalization (OR 6.88, 95% CI 2.48–19.10, *p*<0.001), transfer from another hospital during the index hospitalization (OR 3.45, 95% CI 1.73–6.87, *p*<0.001), and the occurrence of a postoperative event within 90 days after the index surgery (OR 17.76, 95% CI 9.57–32.96, *p*<0.001) were identified as independent factors associated with an elevated risk of unplanned readmission.

### Reoperations

**Fig 2:**
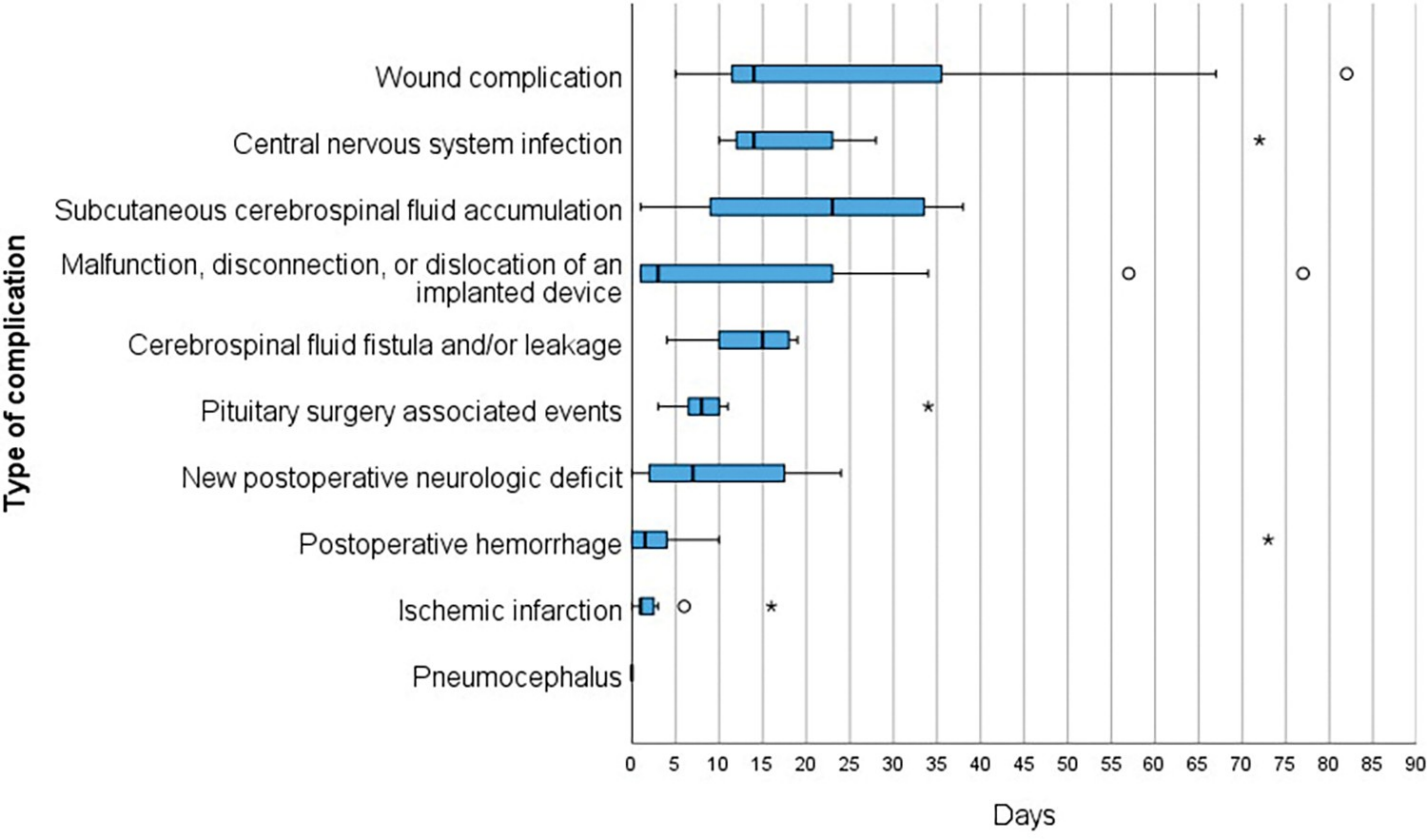
Median number of days until the occurrence of postoperative events.

The 1142 patients in the study cohort underwent a total of 352 reoperations within 90 days after the index surgery. The all-cause 7-, 15-, 30-, 60-, and 90-day reoperation rates were 11.1% (n = 127), 13.8% (n = 158), 16.5% (n = 189), 18.7% (n = 213), and 19.4% (n = 221), respectively. Approximately 56% of the reoperations were unplanned (n = 200, 56.8%). Among the study participants, the rates of unplanned reoperation within 7, 15, 30, 60, and 90 days after the index surgery were 4.6% (n = 53), 6.7% (n = 77), 9.3% (n = 106), 10.6% (n = 121), and 11% (n = 126), respectively. More than three-quarters of reoperations were performed during the index hospitalization (n = 268, 76.1%), and patients required readmission to the Vienna General Hospital in a total of 84 repeated interventions (23.9%). The number of reoperations per patient ranged from 1 to 14. The median durations until planned and unplanned reoperation were 4 days (range: 0–83 days) and 13 days (range: 0–86 days), respectively (Fig 2 in the S1 Appendix).

Unplanned reoperation was most frequently due to hydrocephalus (n = 50, 25%) (pediatric and adult study populations), postoperative hemorrhage (n = 21, 10.5%), or external ventricular drainage-associated complications (n = 18, 9%) (Table 7 in the S1 Appendix). The most common ICD-10 diagnoses at unplanned reoperation were “G91.9 Hydrocephalus, unspecified” (n = 27, 13.5%), “I60.9 Subarachnoid hemorrhage, unspecified” (n = 24, 12%), and “I62.0 Nontraumatic subdural hemorrhage” (n = 11, 5.50%). In unplanned reoperations, the most frequently performed surgeries were “placement of a ventricular shunt” (n = 42, 21%), followed by “placement of an external ventricular drain” (n = 41, 20.50%), and “other operation—neurocranium and dura” (n = 18, 9%).

Multivariate analyses indicated that age ≥90 years (OR 70. 84, 95% CI 2.66–1884.25, *p* = 0.011), urgent index operation (OR 4.77, 95% CI 1.50–15.17, *p* = 0.008), emergent index operation (OR 8.61, 95% CI 1.91–38.82. *p* = 0.005), LOS of 15–21 days at the index hospitalization (OR 13.43, 95% CI 1.63–110.49, *p* = 0.016), LOS of >21 days at the index hospitalization (OR 62.83, 95% CI 7.48–527.50, *p*<0.001), transfer to a rehabilitation center after the index hospitalization (OR 119.57, 95% CI 1.39–10318.09, *p* = 0.035), discharge against medical advice after the index hospitalization (OR 12.31, 95% CI 1.45–104.37, *p* = 0.021), and the occurrence of a postoperative event within 90 days after the index surgery (OR 157.86, 95% CI 56.79–438.76, *p*<0.001) were statistically associated with unplanned reoperation.

### Perioperative events

In the study cohort, 13 patients experienced a total of 15 preoperative events. The overall rate of preoperative events was 1.1%. The duration until the preoperative events was known in 8 of 15 cases, and amounted to a median of 0 days (range: 0–11 days). Approximately three-quarters of the preoperative events occurred in the general ward (n = 11, 73.33%), whereas the rest were reported during intensive care unit or intermediate care unit stays (n = 4, 26.67%). The rate of intraoperative events in the patient cohort was 11% (n = 126). The total number of intraoperative events was 152. Of these, 98 (64.47%) were surgical, 37 (24.34%) were anesthesiologic, and 17 (11.18%) were technical equipment-related complications (Table 9 in the S1 Appendix). No patients died in the operating theater. The rate of postoperative events was 9.5% (n = 109). The most common postoperative events after elective procedures were pituitary surgery associated events (n = 14) and wound complications (n = 13), whereas the malfunction, disconnection, or dislocation of an implanted device was the most frequently described event after non-elective procedures (n = 13) (Table 11 in the S1 Appendix). A time-to-event analysis indicating the average time until the occurrence of postoperative events is shown in Fig 2. All events requiring immediate attention, such as hemorrhage or infarcts, occurred within 4 days after surgery. Factors significantly associated with perioperative events in multivariate logistic regression models are summarized in Table 12 in the S1 Appendix.

### Mortality

The mortality rates within 7, 15, 30, 60, and 90 days after the index operation were 0.9% (n = 10), 1.8% (n = 21), 2.5% (n = 29), 3.4% (n = 39), and 4.7% (n = 54), respectively. The proportion of patients who died within 90 days after the index surgery was significantly higher among patients who underwent an emergency operation (n = 17, 19.1%) (OR 10.11, *p*<0.001) than an elective operation (n = 18, 2.3%). The median time to death was 25 days (range: 1–90 days). The number of female and male patients who died was equal (n = 27, 50%). More than half of the deaths occurred after patient discharge from the index hospitalization (n = 29, 53.7%), approximately one-third of the patients died in the intensive care unit or intermediate care unit (n = 18, 33.33%), and 12.96% of the patients died in the general ward (n = 7). the intraoperative mortality rate was 0% (n = 0). Kaplan-Meier curves depicting the time to death between patients with or without extended LOS, 90-day readmission, or 90-day reoperation are presented in Figs 3–5 in the S1 Appendix, respectively.

## Discussion

Regular measurement and analysis of outcome parameters is crucial in evaluating the quality of healthcare systems and enabling necessary steps toward improvement.

In our study, the median LOS at the index hospitalization was 8 days, which was longer than the reported neurosurgical hospitalizations in Anglo-American countries [33, 34]. Approximately one-fifth of patients had an extended LOS of at least 14 days. Our findings highlighted that, despite an observed slow decline in LOS in the nine neurosurgical departments in Austria over the past two decades [35], further initiatives are necessary to avoid hospital days beyond medical readiness [36].

The main reason for the discrepancy in the LOS between European countries such as Austria and countries such as the United States of America lies in historically deep-seated cultural differences regarding when patient discharge is considered safe by physicians and patients, in the absence of any data supporting that longer hospital stays increase safety. Acquiring evidence-based data is the only way to change the perceptions and traditions that have resulted in patients remaining in hospitals until their stiches have been removed, as reflected by the median hospital stay of 8 days. Our study provides evidence that patients without identified risk factors—most importantly neurological compromise, diminished albumin, elevated prothrombin time at admission, and intraoperative events—could be discharged earlier in the health care environment of Austria. All early complications requiring immediate attention, such as postoperative hemorrhage or infarcts, occurred within 4 days after surgery. All late complications seen after discharge were readily identified in the outpatient setting, and were associated primarily with wound complications and infections.

Furthermore, to decrease the LOS among neurosurgical patients in Austria, special attention must be paid to patients after transphenoidal pituitary surgery, given that a substantial number of events associated with hyponatremia may potentially be life-threatening. Similarly, diminished levels of potassium in patients with a cardiac comorbidity should be normalized before admission. Steroid use in patients admitted for brain tumor surgery should be critically assessed for reduction or termination, if possible, given that unnecessary steroid prescription is associated with an increased complication rate and extended hospital stay [37–40].

In addition, a concomitant decrease in preventable unplanned readmission, thus avoiding unnecessary costs, can be accomplished only if the risk factors for readmission have been identified. Herein, we identified that a preoperative sodium level < 135 mm/L, prior deep vein thrombosis, the presence of psychosis, and transfer from another department or hospital were independent risk factors for unplanned readmission among neurosurgical patients. With regard to unplanned reoperation, we performed a detailed analysis and identified that age ≥90 years, urgent or emergent index operation, extended LOS, and transfer to a rehabilitation center after index hospitalization, among other factors, were statistically associated with unplanned reoperation.

All the above-identified factors must be considered when regulatory measures to decrease the LOS in neurosurgery are developed in health care policies. In Austria, increased resource allocation for outpatient care would be necessary to provide a safe environment for neurosurgical patients when the LOS is further decreased [38]. Similarly, for optimization of preoperative outpatient care, we identified a preoperative in-hospital event rate of 1.1%. Most in-hospital adverse events occurring before the index surgery were associated with infection. Our results emphasize that patients are at risk of complications even before they undergo surgery; therefore unnecessary inpatient days before surgery, as well as surgical delays after hospital admission, should be avoided. We also identified that several abnormalities in preoperative laboratory values were associated with preoperative events. These findings suggest that patients who are predisposed to complications could be identified, and their clinical condition could be optimized before hospital admission for an elective neurosurgical operation.

Finally, our study provides a definition of postoperative events that could be used for future comparative analyses of neurosurgical patient populations, by defining a postoperative adverse events as any abnormal or unexpected neurosurgical event occurring within 90 days after the index surgery, and contributing to a prolonged hospitalization, readmission, or reoperation [7, 41–44]. In our cohort, the proportion of patients who experienced a postoperative event, according to this definition, was 8.4% (n = 96).

### Strengths and limitations

The major limitation of our study is its single-center design, which limits the generalizability of our results to other centers in Austria. Although a comparison with the three other large academic centers in Austria might have increased the reliability of the findings, given that patients with the most complex cases requiring comprehensive care are often referred to those centers, the selection bias toward less complex surgeries in the five regional hospitals might have been too pronounced to enable a detailed analysis. However, by taking the unspecific measure of the LOS into account, clear parallels can be seen, thereby allowing for an indirect extrapolation [35]. Furthermore, because Austria is a small country with no more than 9 million inhabitants, any major differences in patient care across the nine neurosurgical centers, which could potentially have greatly influenced the outcome parameters analyzed herein, would be expected to be small, but nonetheless must be critically addressed. Furthermore, the study period’s spanning January through December of 2020, in the midst of the COVID-19 pandemic, must be critically considered. In contrast to many other neurosurgical departments worldwide, patient care at our institution was not affected by the COVID-19 pandemic to an extent that could have made our study population not representative in this specific regard [45]. Among the multifactorial reasons for the lack of influence of the pandemic at our institution, the most important reason was that our neurosurgical department is situated in a separate building from the main hospital, where all the other departments are located. Therefore, neither the neurosurgical ICU nor our general ward’s bed capacity was affected by any restrictions during the study period. Indeed, the overall case load was slightly greater than that in previous years, thus indicating that the any limitations due to the study period itself might have been counterintuitively small.

Simultaneously, our analysis encompassed the entire spectrum of neurosurgical diseases and procedures across all age groups. Furthermore, because our analyses were not based solely on administrative datasets with coded information, we were able to collect extensive and precise information on patient characteristics, perioperative factors, and outcomes. Nonetheless, the descriptions of intraoperative events in this study were restricted to the reports of the operating surgeons.

### Conclusion

This study identified risk factors for prolonged LOS, readmission, and reoperation in a diverse neurosurgical patient cohort in Europe. The findings highlight LOS among neurosurgical patients in Austria could feasibly be decreased if outpatient care were tailored to the specific needs of this vulnerable patient population.

## Supporting information

S1 Appendix (DOCX)

## Abbreviations

AIDS: Acquired immune deficiency syndrome
ASA: American Society of Anesthesiology
BMI: Body mass index
ER: Emergency room
HIV: Human immunodeficiency virus
ICD-10: International Classification of Diseases 10th Version
ICU: Intensive care unit
LOS: Length of hospital stay
NSAID: Non-steroidal anti-inflammatory drug
WHO: World Health Organization
