# Supplementary material for: Perioperative quality indicators among neurosurgery patients: A retrospective cohort study of 1142 cases at a tertiary center
Source: PLoS One. 2024 Feb 6;19(2):e0297167. doi: 10.1371/journal.pone.0297167 (PMC10846709; doi:10.1371/journal.pone.0297167)
Supplement: S1 Appendix — (DOCX) [file pone.0297167.s001.docx]

**Perioperative quality indicators among neurosurgery patients: A retrospective cohort study of 1142 cases at a tertiary center**

Edzhem Chavush^1^, Karl Rössler^1^, Christian Dorfer^1*^

1 Department of Neurosurgery, Medical University of Vienna, Vienna, Austria.

* Corresponding author

E-Mail: christian.dorfer@meduniwien.ac.at

**Supplementary Appendix 1**

**Tables**

**Table 1.** Results of perioperative quality indicators stratified by preoperative laboratory values

|  | **No. of patients Total=1142** | | **LOS at Index Admission (in days)** | **Proportion of Patients with an Extended LOS** | | **90-Day Unplanned Readmission Rate** | | **90-Day Unplanned Reoperation Rate** | | **Preoperative Event Rate** | | **Intraoperative Event Rate** | | **Postoperative Event Rate** | | **90-Day Postoperative Mortality Rate** | |
| --- | --- | --- | --- | --- | --- | --- | --- | --- | --- | --- | --- | --- | --- | --- | --- | --- | --- |
| **Variables** | **N** | **%** | **Median** | **%** | ***p*-value** | **%** | ***p*-value** | **%** | ***p*-value** | **%** | ***p*-value** | **%** | ***p*-value** | **%** | ***p*-value** | **%** | ***p*-value** |
| Preoperative laboratory values |  |  |  |  |  |  |  |  |  |  |  |  |  |  |  |  |  |
| Sodium (mmol/L) |  |  |  |  |  |  |  |  |  |  |  |  |  |  |  |  |  |
| <135 | 30 | 2.6 | 10.5 | 33.3 | 0.119 | 20.0 | 0.034 | 10.0 | 0.832 | 6.7 | 0.029 | 6.7 | 0.516 | 13.3 | 0.423 | 10.0 | 0.154 |
| 135-145 | 811 | 71.0 | 9.0 | 21.2 | Ref. | 8.4 | Ref. | 8.9 | Ref. | 1.2 | Ref. | 10.4 | Ref. | 9.0 | Ref. | 4.3 | Ref. |
| >145 | 17 | 1.5 | 11.0 | 41.2 | 0.056 | 5.9 | 0.713 | 23.5 | 0.049 | 5.9 | 0.135 | 11.8 | 0.851 | 11.8 | 0.695 | 11.8 | 0.161 |
| Not measured | 284 | 24.9 | 8.0 | 25.7 | 0.118 | 7.7 | 0.736 | 16.5 | <0.001 | 0.0 | 0.994 | 13.4 | 0.165 | 10.6 | 0.438 | 4.9 | 0.667 |
| Potassium (mmol/L) |  |  |  |  |  |  |  |  |  |  |  |  |  |  |  |  |  |
| <3.5 | 40 | 3.5 | 13.5 | 50.0 | <0.001 | 0.0 | 0.998 | 27.5 | <0.001 | 2.5 | 0.554 | 12.5 | 0.608 | 20.0 | 0.020 | 10.0 | 0.118 |
| 3.5-5.5 | 741 | 64.9 | 9.0 | 20.2 | Ref. | 9.4 | Ref. | 8.2 | Ref. | 1.3 | Ref. | 10.0 | Ref. | 8.6 | Ref. | 4.5 | Ref. |
| >5.5 | 4 | 0.4 | 10.0 | 25.0 | 0.814 | 0.0 | 0.999 | 25.0 | 0.259 | 0.0 | 0.999 | 25.0 | 0.343 | 25.0 | 0.278 | 0.0 | 0.999 |
| Not measured | 357 | 31.3 | 8.0 | 25.5 | 0.050 | 7.6 | 0.304 | 14.8 | <0.001 | 0.6 | 0.254 | 12.9 | 0.150 | 10.1 | 0.435 | 4.8 | 0.818 |
| Chloride (mmol/L) |  |  |  |  |  |  |  |  |  |  |  |  |  |  |  |  |  |
| <95 | 12 | 1.1 | 8.0 | 16.7 | 0.804 | 16.7 | 0.380 | 16.7 | 0.368 | 8.3 | 0.079 | 8.3 | 0.887 | 8.3 | 0.899 | 16.7 | 0.063 |
| 95-105 | 691 | 60.5 | 8.0 | 19.5 | Ref. | 9.1 | Ref. | 9.0 | Ref. | 1.3 | Ref. | 9.6 | Ref. | 9.4 | Ref | 4.3 | Ref. |
| >105 | 150 | 13.1 | 9.0 | 33.3 | <0.001 | 6.7 | 0.336 | 10.0 | 0.693 | 2.0 | 0.517 | 14.0 | 0.107 | 8.7 | 0.777 | 5.3 | 0.597 |
| Not measured | 289 | 25.3 | 8.0 | 26.0 | 0.026 | 7.6 | 0.446 | 16.3 | 0.001 | 0.0 | 0.994 | 13.1 | 0.097 | 10.4 | 0.639 | 4.8 | 0.729 |
| Creatinine (mg/dL) |  |  |  |  |  |  |  |  |  |  |  |  |  |  |  |  |  |
| <1.2 | 873 | 76.4 | 9.0 | 23.6 | Ref. | 9.2 | Ref. | 10.9 | Ref. | 1.4 | Ref. | 9.9 | Ref. | 10.0 | Ref. | 4.5 | Ref. |
| ≥1.2 | 40 | 3.5 | 9.5 | 35.0 | 0.103 | 7.5 | 0.721 | 10.0 | 0.861 | 2.5 | 0.563 | 15.0 | 0.294 | 5.0 | 0.311 | 17.5 | <0.001 |
| Not measured | 229 | 20.1 | 7.0 | 18.3 | 0.091 | 6.1 | 0.144 | 11.8 | 0.697 | 0.0 | 0.995 | 14.8 | 0.032 | 8.7 | 0.575 | 3.5 | 0.517 |
| Albumin (g/L) |  |  |  |  |  |  |  |  |  |  |  |  |  |  |  |  |  |
| <35 | 59 | 5.2 | 19.0 | 67.8 | <0.001 | 10.2 | 0.850 | 32.2 | <0.001 | 5.1 | 0.034 | 18.6 | 0.037 | 15.3 | 0.191 | 13.6 | 0.002 |
| 35-50 | 722 | 63.2 | 9.0 | 22.0 | Ref. | 9.4 | Ref. | 9.6 | Ref. | 1.2 | Ref. | 9.8 | Ref. | 9.8 | Ref. | 4.0 | Ref. |
| >50 | 26 | 2.3 | 8.5 | 7.7 | 0.100 | 7.7 | 0.767 | 7.7 | 0.751 | 0.0 | 0.998 | 11.5 | 0.775 | 7.7 | 0.719 | 0.0 | 0.998 |
| Not measured | 335 | 29.3 | 7.0 | 18.2 | 0.156 | 6.3 | 0.088 | 10.7 | 0.548 | 0.3 | 0.173 | 12.2 | 0.238 | 8.1 | 0.356 | 5.1 | 0.434 |
| White blood cell count (G/L) |  |  |  |  |  |  |  |  |  |  |  |  |  |  |  |  |  |
| <4 | 18 | 1.6 | 7.5 | 11.1 | 0.485 | 5.6 | 0.673 | 5.6 | 0.717 | 5.6 | 0.156 | 0.0 | 0.998 | 0.0 | 0.998 | 11.1 | 0.082 |
| 4-10 | 646 | 56.6 | 8.0 | 17.5 | Ref. | 8.4 | Ref. | 7.9 | Ref. | 1.2 | Ref. | 9.8 | Ref. | 8.0 | Ref. | 3.1 | Ref. |
| >10 | 252 | 22.1 | 11.0 | 41.7 | <0.001 | 10.7 | 0.269 | 18.3 | <0.001 | 1.6 | 0.683 | 11.5 | 0.436 | 14.7 | 0.003 | 9.1 | <0.001 |
| Not measured | 226 | 19.8 | 7.0 | 18.6 | 0.712 | 6.6 | 0.410 | 12.4 | 0.044 | 0.0 | 0.995 | 15.0 | 0.031 | 8.8 | 0.707 | 4.0 | 0.524 |
| Hematocrit (%) |  |  |  |  |  |  |  |  |  |  |  |  |  |  |  |  |  |
| <30 | 33 | 2.9 | 11.0 | 39.4 | 0.199 | 15.2 | 0.323 | 24.2 | 0.064 | 6.1 | 0.086 | 9.1 | 0.578 | 12.1 | 0.594 | 18.2 | 0.006 |
| 30-40 | 452 | 39.6 | 9.0 | 28.8 | Ref. | 9.7 | Ref. | 12.6 | Ref. | 1.5 | Ref. | 12.4 | Ref. | 9.3 | Ref. | 5.3 | Ref. |
| >40 | 429 | 37.6 | 8.0 | 17.9 | <0.001 | 7.5 | 0.231 | 7.7 | 0.017 | 0.9 | 0.415 | 7.7 | 0.022 | 10.0 | 0.713 | 3.5 | 0.194 |
| Not measured | 228 | 20.0 | 7.0 | 18.4 | 0.004 | 7.0 | 0.240 | 12.3 | 0.902 | 0.0 | 0.995 | 14.9 | 0.360 | 8.8 | 0.824 | 3.9 | 0.437 |
| Platelet count (G/L) |  |  |  |  |  |  |  |  |  |  |  |  |  |  |  |  |  |
| <150 | 29 | 2.5 | 10.0 | 44.8 | 0.008 | 17.2 | 0.105 | 17.2 | 0.209 | 0.0 | 0.998 | 13.8 | 0.517 | 3.4 | 0.265 | 27.6 | <0.001 |
| 150-350 | 765 | 67.0 | 9.0 | 22.6 | Ref. | 8.4 | Ref. | 9.9 | Ref. | 1.3 | Ref. | 10.1 | Ref. | 10.1 | Ref. | 4.2 | Ref. |
| >350 | 121 | 10.6 | 9.0 | 28.1 | 0.186 | 10.7 | 0.390 | 14.0 | 0.172 | 2.5 | 0.327 | 9.1 | 0.739 | 9.1 | 0.739 | 4.1 | 0.979 |
| Not measured | 227 | 19.9 | 7.0 | 18.5 | 0.187 | 6.6 | 0.391 | 12.3 | 0.301 | 0.0 | 0.995 | 15.0 | 0.040 | 8.8 | 0.576 | 4.0 | 0.885 |
| Prothrombin time (Owren) (%) |  |  |  |  |  |  |  |  |  |  |  |  |  |  |  |  |  |
| <70 | 95 | 8.3 | 12.0 | 47.4 | <0.001 | 13.7 | 0.071 | 25.3 | <0.001 | 4.2 | 0.025 | 10.5 | 0.975 | 18.9 | 0.005 | 10.5 | 0.011 |
| 70-125 | 743 | 65.1 | 9.0 | 20.6 | Ref. | 8.1 | Ref. | 9.4 | Ref. | 1.1 | Ref. | 10.6 | Ref. | 9.4 | Ref. | 4.3 | Ref. |
| >125 | 63 | 5.5 | 7.0 | 20.6 | 0.994 | 7.9 | 0.969 | 6.3 | 0.421 | 1.6 | 0.713 | 6.3 | 0.289 | 4.8 | 0.226 | 1.6 | 0.316 |
| Not measured | 241 | 21.1 | 7.0 | 21.2 | 0.850 | 7.9 | 0.924 | 11.6 | 0.323 | 0.0 | 0.995 | 13.7 | 0.195 | 7.5 | 0.357 | 4.6 | 0.865 |
| International normalized ratio |  |  |  |  |  |  |  |  |  |  |  |  |  |  |  |  |  |
| <1.5 | 897 | 78.5 | 9.0 | 23.5 | Ref. | 8.6 | Ref. | 10.6 | Ref. | 1.4 | Ref. | 10.3 | Ref. | 10.0 | Ref. | 4.6 | Ref. |
| ≥1.5 | 9 | 0.8 | 7.0 | 22.2 | 0.927 | 22.2 | 0.170 | 33.3 | 0.044 | 0.0 | 0.999 | 11.1 | 0.933 | 11.1 | 0.915 | 33.3 | 0.001 |
| Not measured | 236 | 20.7 | 7.0 | 20.8 | 0.370 | 7.6 | 0.637 | 11.9 | 0.576 | 0.0 | 0.995 | 14.0 | 0.105 | 7.6 | 0.264 | 4.2 | 0.826 |
| Activated partial thromboplastin time (s) |  |  |  |  |  |  |  |  |  |  |  |  |  |  |  |  |  |
| <27 | 28 | 2.5 | 10.5 | 28.6 | 0.475 | 3.6 | 0.323 | 3.6 | 0.262 | 0.0 | 0.998 | 14.3 | 0.537 | 14.3 | 0.432 | 10.7 | 0.108 |
| 27-41 | 821 | 71.9 | 9.0 | 22.8 | Ref. | 9.3 | Ref. | 10.5 | Ref. | 1.5 | Ref. | 10.6 | Ref. | 9.7 | Ref. | 4.1 | Ref. |
| >41 | 59 | 5.2 | 9.0 | 32.2 | 0.101 | 5.1 | 0.287 | 18.6 | 0.057 | 1.7 | 0.886 | 3.4 | 0.095 | 11.9 | 0.599 | 11.9 | 0.010 |
| Not measured | 234 | 20.5 | 7.00 | 20.5 | 0.463 | 7.3 | 0.344 | 12.0 | 0.517 | 0.0 | 0.995 | 14.1 | 0.138 | 7.7 | 0.341 | 4.3 | 0.929 |
| Fibrinogen (Clauss) (mg/dL) |  |  |  |  |  |  |  |  |  |  |  |  |  |  |  |  |  |
| <200 | 38 | 3.3 | 9.0 | 31.6 | 0.103 | 7.9 | 0.823 | 13.2 | 0.656 | 0.0 | 0.998 | 7.9 | 0.658 | 15.8 | 0.214 | 10.5 | 0.059 |
| 200-400 | 692 | 60.6 | 9.0 | 20.4 | Ref. | 9.0 | Ref. | 10.8 | Ref. | 1.3 | Ref. | 10.1 | Ref. | 9.5 | Ref. | 3.9 | Ref. |
| >400 | 172 | 15.1 | 9.0 | 33.7 | <0.001 | 7.6 | 0.560 | 10.5 | 0.888 | 2.3 | 0.330 | 11.6 | 0.562 | 11.0 | 0.552 | 7.0 | 0.086 |
| Not measured | 240 | 21.0 | 7.5 | 21.3 | 0.773 | 7.9 | 0.621 | 11.7 | 0.724 | 0.0 | 0.995 | 13.8 | 0.123 | 7.5 | 0.343 | 4.6 | 0.646 |
| Blood urea nitrogen (BUN) (mg/dL) |  |  |  |  |  |  |  |  |  |  |  |  |  |  |  |  |  |
| <6 | 23 | 2.0 | 9.0 | 34.8 | 0.207 | 0.0 | 0.998 | 8.7 | 0.759 | 4.3 | 0.185 | 13.0 | 0.601 | 4.3 | 0.386 | 4.3 | 0.960 |
| 6-25 | 832 | 72.9 | 9.0 | 23.3 | Ref. | 9.0 | Ref. | 10.7 | Ref. | 1.1 | Ref. | 9.7 | Ref. | 10.0 | Ref. | 4.6 | Ref. |
| >25 | 48 | 4.2 | 9.5 | 33.3 | 0.117 | 14.6 | 0.202 | 16.7 | 0.204 | 6.3 | 0.008 | 16.7 | 0.127 | 10.4 | 0.921 | 12.5 | 0.019 |
| Not measured | 239 | 20.9 | 7.0 | 18.4 | 0.109 | 6.3 | 0.181 | 11.3 | 0.793 | 0.0 | 0.995 | 14.2 | 0.049 | 8.4 | 0.458 | 3.8 | 0.594 |
| Glutamic oxaloacetic transaminase (GOT) (U/L) |  |  |  |  |  |  |  |  |  |  |  |  |  |  |  |  |  |
| <35 | 658 | 57.6 | 9.0 | 26.1 | Ref. | 9.1 | Ref. | 11.4 | Ref. | 1.4 | Ref. | 9.3 | Ref. | 10.9 | Ref. | 4.1 | Ref. |
| ≥35 | 153 | 13.4 | 9.0 | 24.8 | 0.740 | 7.8 | 0.618 | 9.8 | 0.572 | 2.0 | 0.586 | 15.7 | 0.021 | 7.2 | 0.171 | 7.8 | 0.056 |
| Not measured | 331 | 29.0 | 7.0 | 15.7 | <0.001 | 7.6 | 0.408 | 10.9 | 0.806 | 0.3 | 0.150 | 12.4 | 0.130 | 7.9 | 0.127 | 4.5 | 0.753 |
| Glutamate-pyruvate transaminase (GPT) (U/L) |  |  |  |  |  |  |  |  |  |  |  |  |  |  |  |  |  |
| <40 | 685 | 60.0 | 9.0 | 25.3 | Ref. | 9.6 | Ref. | 12.6 | Ref. | 1.3 | Ref. | 10.7 | Ref. | 10.5 | Ref. | 5.3 | Ref. |
| ≥40 | 151 | 13.2 | 8.0 | 25.8 | 0.884 | 6.0 | 0.157 | 5.3 | 0.013 | 2.0 | 0.532 | 10.6 | 0.982 | 8.6 | 0.485 | 4.0 | 0.515 |
| Not measured | 306 | 26.8 | 7.0 | 16.3 | 0.002 | 7.2 | 0.213 | 10.5 | 0.347 | 0.3 | 0.185 | 12.1 | 0.507 | 7.8 | 0.191 | 3.9 | 0.368 |
| Lactate dehydrogenase (LDH) (U/L) |  |  |  |  |  |  |  |  |  |  |  |  |  |  |  |  |  |
| <125 | 31 | 2.7 | 9.0 | 32.3 | 0.335 | 6.5 | 0.717 | 16.1 | 0.328 | 0.0 | 0.998 | 6.5 | 0.507 | 16.1 | 0.294 | 0.0 | 0.998 |
| 125-250 | 591 | 51.8 | 9.0 | 24.5 | Ref. | 8.3 | Ref. | 10.5 | Ref. | 1.5 | Ref. | 10.2 | Ref. | 10.2 | Ref. | 4.4 | Ref. |
| >250 | 134 | 11.7 | 10.0 | 29.9 | 0.203 | 11.2 | 0.287 | 13.4 | 0.328 | 1.5 | 0.979 | 14.2 | 0.179 | 9.0 | 0.676 | 9.0 | 0.036 |
| Not measured | 386 | 33.8 | 7.0 | 17.4 | 0.008 | 8.0 | 0.885 | 10.6 | 0.948 | 0.5 | 0.165 | 11.7 | 0.458 | 8.3 | 0.331 | 4.1 | 0.848 |
| Triglyceride (mg/dL) |  |  |  |  |  |  |  |  |  |  |  |  |  |  |  |  |  |
| <150 | 482 | 42.2 | 8.0 | 22.2 | Ref. | 8.5 | Ref. | 11.0 | Ref. | 1.5 | Ref. | 10.0 | Ref. | 10.8 | Ref. | 5.0 | Ref. |
| ≥150 | 197 | 17.3 | 9.0 | 24.9 | 0.452 | 8.1 | 0.870 | 9.6 | 0.604 | 1.0 | 0.653 | 11.2 | 0.638 | 10.2 | 0.807 | 5.6 | 0.747 |
| Not measured | 463 | 40.5 | 9.0 | 22.9 | 0.798 | 8.6 | 0.942 | 11.7 | 0.746 | 0.9 | 0.404 | 12.1 | 0.295 | 8.0 | 0.142 | 4.1 | 0.519 |
| Cholesterol (total) (mg/dL) |  |  |  |  |  |  |  |  |  |  |  |  |  |  |  |  |  |
| <200 | 387 | 33.9 | 8.0 | 22.2 | Ref. | 7.8 | Ref. | 8.8 | Ref. | 1.8 | Ref. | 8.3 | Ref. | 9.3 | Ref. | 4.9 | Ref. |
| ≥200 | 204 | 17.9 | 8.0 | 14.7 | 0.030 | 8.8 | 0.650 | 8.3 | 0.852 | 0.0 | 0.995 | 10.3 | 0.413 | 11.3 | 0.448 | 2.0 | 0.089 |
| Not measured | 551 | 48.2 | 9.0 | 26.5 | 0.136 | 8.9 | 0.536 | 13.6 | 0.024 | 1.1 | 0.358 | 13.2 | 0.018 | 9.1 | 0.905 | 5.6 | 0.631 |
| C-reactive protein (mg/dL) |  |  |  |  |  |  |  |  |  |  |  |  |  |  |  |  |  |
| <0.5 | 704 | 61.6 | 8.5 | 20.6 | Ref. | 9.4 | Ref. | 9.7 | Ref. | 0.9 | Ref. | 9.9 | Ref. | 10.5 | Ref. | 4.0 | Ref. |
| ≥0.5 | 208 | 18.2 | 10.0 | 36.1 | <0.001 | 7.2 | 0.337 | 14.9 | 0.034 | 3.4 | 0.013 | 10.6 | 0.790 | 7.2 | 0.161 | 8.7 | 0.008 |
| Not measured | 230 | 20.1 | 7.0 | 18.3 | 0.442 | 7.0 | 0.262 | 11.7 | 0.366 | 0.0 | 0.995 | 14.8 | 0.044 | 8.7 | 0.428 | 3.5 | 0.733 |
| HbA1c (%) |  |  |  |  |  |  |  |  |  |  |  |  |  |  |  |  |  |
| <6 | 457 | 40.0 | 8.0 | 12.9 | Ref. | 7.2 | Ref. | 6.8 | Ref. | 0.7 | Ref. | 10.5 | Ref. | 8.5 | Ref. | 2.4 | Ref. |
| ≥6 | 91 | 8.0 | 9.0 | 18.7 | 0.148 | 12.1 | 0.123 | 7.7 | 0.755 | 2.2 | 0.183 | 11.0 | 0.891 | 12.1 | 0.285 | 5.5 | 0.120 |
| Not measured | 594 | 52.0 | 9.0 | 31.3 | <0.001 | 8.9 | 0.319 | 14.8 | <0.001 | 1.3 | 0.286 | 11.4 | 0.628 | 9.9 | 0.440 | 6.4 | 0.003 |

**Table 2.** Assessments of additional patient characteristics and clinical parameters

|  | **No. of patients Total=1142** | | **LOS at Index Admission (in days)** | **Proportion of Patients with an Extended LOS** | | **90-Day Unplanned Readmission Rate** | | **90-Day Unplanned Reoperation Rate** | | **Preoperative Event Rate** | | **Intraoperative Event Rate** | | **Postoperative Event Rate** | | **90-Day Postoperative Mortality Rate** | |
| --- | --- | --- | --- | --- | --- | --- | --- | --- | --- | --- | --- | --- | --- | --- | --- | --- | --- |
| **Variables** | **N** | **%** | **Median** | **%** | ***p*-value** | **%** | ***p*-value** | **%** | ***p*-value** | **%** | ***p*-value** | **%** | ***p*-value** | **%** | ***p*-value** | **%** | ***p*-value** |
| Ability to perform daily activities in home environment (n=980) |  |  |  |  |  |  |  |  |  |  |  |  |  |  |  |  |  |
| Yes | 856 | 87.3 | 8.0 | 16.6 | Ref. | 7.4 | Ref. | 8.6 | Ref. | 0.7 | Ref. | 10.6 | Ref. | 9.0 | Ref. | 3.3 | Ref. |
| No | 44 | 4.5 | 9.5 | 45.5 | <0.001 | 6.8 | 0.893 | 25.0 | <0.001 | 2.3 | 0.275 | 11.4 | 0.878 | 13.6 | 0.304 | 15.9 | <0.001 |
| Unknown | 80 | 8.2 | 15.5 | 53.8 | <0.001 | 12.5 | 0.106 | 18.8 | 0.004 | 1.3 | 0.591 | 13.8 | 0.393 | 13.8 | 0.167 | 18.8 | <0.001 |
| Living situation (n=980) |  |  |  |  |  |  |  |  |  |  |  |  |  |  |  |  |  |
| Without support | 852 | 86.9 | 8.0 | 18.7 | Ref. | 7.0 | Ref. | 9.7 | Ref. | 0.8 | Ref. | 10.8 | Ref. | 9.6 | Ref. | 3.5 | Ref. |
| With support | 70 | 7.1 | 9.5 | 25.7 | 0.152 | 12.9 | 0.080 | 12.9 | 0.405 | 0.0 | 0.997 | 11.4 | 0.870 | 10.0 | 0.919 | 11.4 | 0.003 |
| Unknown | 58 | 5.9 | 12.5 | 48.3 | <0.001 | 12.1 | 0.162 | 13.8 | 0.323 | 1.7 | 0.486 | 12.1 | 0.764 | 8.6 | 0.802 | 20.7 | <0.001 |
| Day of index admission to the hospital |  |  |  |  | 0.830 |  | 0.345 |  | 0.613 |  | 0.745 |  | 0.024 |  | 0.128 |  | 0.612 |
| Weekday | 899 | 78.7 | 8.0 | 22.8 |  | 8.9 |  | 10.8 |  | 1.1 |  | 12.1 |  | 10.2 |  | 4.9 |  |
| Weekend | 243 | 21.3 | 8.0 | 23.5 |  | 7.0 |  | 11.9 |  | 1.2 |  | 7.0 |  | 7.0 |  | 4.1 |  |
| Month of index admission to the hospital |  |  |  |  | 0.194 |  | 0.327 |  | 0.057 |  | 0.511 |  | 0.032 |  | 0.216 |  | 0.662 |
| January | 104 | 9.1 | 9.0 | 25.0 |  | 9.6 |  | 10.6 |  | 1.9 |  | 9.6 |  | 9.6 |  | 2.9 |  |
| February | 104 | 9.1 | 9.0 | 25.0 |  | 6.7 |  | 14.4 |  | 2.9 |  | 15.4 |  | 8.7 |  | 5.8 |  |
| March | 78 | 6.8 | 8.0 | 16.7 |  | 5.1 |  | 6.4 |  | 0.0 |  | 5.1 |  | 5.1 |  | 1.3 |  |
| April | 69 | 6.0 | 9.0 | 30.4 |  | 8.7 |  | 7.2 |  | 0.0 |  | 11.6 |  | 8.7 |  | 10.1 |  |
| May | 88 | 7.7 | 9.0 | 26.1 |  | 8.0 |  | 13.6 |  | 1.1 |  | 12.5 |  | 14.8 |  | 3.4 |  |
| June | 102 | 8.9 | 8.0 | 28.4 |  | 7.8 |  | 12.7 |  | 1.0 |  | 15.7 |  | 10.8 |  | 3.9 |  |
| July | 98 | 8.6 | 8.0 | 22.4 |  | 12.2 |  | 18.4 |  | 2.0 |  | 8.2 |  | 16.3 |  | 5.1 |  |
| August | 115 | 10.1 | 8.0 | 24.3 |  | 10.4 |  | 7.8 |  | 0.0 |  | 4.3 |  | 7.8 |  | 4.3 |  |
| September | 103 | 9.0 | 9.0 | 23.3 |  | 12.6 |  | 14.6 |  | 1.9 |  | 9.7 |  | 7.8 |  | 4.9 |  |
| October | 92 | 8.1 | 8.0 | 23.9 |  | 3.3 |  | 13.0 |  | 0.0 |  | 15.2 |  | 12.0 |  | 4.3 |  |
| November | 103 | 9.0 | 8.0 | 17.5 |  | 4.9 |  | 3.9 |  | 1.9 |  | 17.5 |  | 4.9 |  | 6.8 |  |
| December | 86 | 7.5 | 7.0 | 11.6 |  | 11.6 |  | 8.1 |  | 0.0 |  | 7.0 |  | 8.1 |  | 4.7 |  |
| Timing of the hospital admission with regard to the COVID-19 pandemic |  |  |  |  | 0.998 |  | 0.655 |  | 0.641 |  | 0.043 |  | 0.961 |  | 0.687 |  | 0.804 |
| Pre-COVID-19 pandemic | 183 | 16.0 | 9.0 | 23.0 |  | 7.7 |  | 12.0 |  | 2.7 |  | 10.9 |  | 8.7 |  | 4.4 |  |
| Post-COVID-19 pandemic | 959 | 84.0 | 8.0 | 22.9 |  | 8.7 |  | 10.8 |  | 0.8 |  | 11.1 |  | 9.7 |  | 4.8 |  |
| Month of index operation |  |  |  |  | 0.456 |  | 0.326 |  | 0.161 |  | 0.675 |  | 0.005 |  | 0.151 |  | 0.702 |
| January | 99 | 8.7 | 9.0 | 23.2 |  | 10.1 |  | 10.1 |  | 2.0 |  | 9.1 |  | 10.1 |  | 3.0 |  |
| February | 103 | 9.0 | 9.0 | 22.3 |  | 5.8 |  | 13.6 |  | 2.9 |  | 14.6 |  | 6.8 |  | 4.9 |  |
| March | 81 | 7.1 | 8.0 | 23.5 |  | 4.9 |  | 7.4 |  | 0.0 |  | 7.4 |  | 6.2 |  | 2.5 |  |
| April | 70 | 6.1 | 9.0 | 27.1 |  | 8.6 |  | 8.6 |  | 0.0 |  | 10.0 |  | 10.0 |  | 10.0 |  |
| May | 86 | 7.5 | 9.0 | 25.6 |  | 9.3 |  | 14.0 |  | 1.2 |  | 14.0 |  | 14.0 |  | 3.5 |  |
| June | 100 | 8.8 | 9.0 | 30.0 |  | 8.0 |  | 13.0 |  | 1.0 |  | 16.0 |  | 12.0 |  | 4.0 |  |
| July | 102 | 8.9 | 8.0 | 22.5 |  | 11.8 |  | 16.7 |  | 2.0 |  | 7.8 |  | 15.7 |  | 4.9 |  |
| August | 109 | 9.5 | 8.0 | 22.9 |  | 10.1 |  | 9.2 |  | 0.0 |  | 3.7 |  | 8.3 |  | 4.6 |  |
| September | 102 | 8.9 | 9.0 | 22.5 |  | 11.8 |  | 12.7 |  | 2.0 |  | 8.8 |  | 6.9 |  | 2.9 |  |
| October | 96 | 8.4 | 8.0 | 26.0 |  | 5.2 |  | 14.6 |  | 0.0 |  | 14.6 |  | 12.5 |  | 6.3 |  |
| November | 96 | 8.4 | 7.0 | 13.5 |  | 3.1 |  | 3.1 |  | 1.0 |  | 20.8 |  | 3.1 |  | 7.3 |  |
| December | 98 | 8.6 | 7.0 | 17.3 |  | 12.2 |  | 8.2 |  | 1.0 |  | 6.1 |  | 9.2 |  | 4.1 |  |
| Timing of the index operation with regard to the COVID-19 pandemic |  |  |  |  | 0.977 |  | 0.799 |  | 0.480 |  | 0.036 |  | 0.856 |  | 0.634 |  | 0.915 |
| Pre-COVID-19 pandemic | 175 | 15.3 | 9.0 | 22.9 |  | 8.0 |  | 12.6 |  | 2.9 |  | 11.4 |  | 8.6 |  | 4.6 |  |
| Post-COVID-19 pandemic | 967 | 84.7 | 8.0 | 23.0 |  | 8.6 |  | 10.8 |  | 0.8 |  | 11.0 |  | 9.7 |  | 4.8 |  |
| Extended length of hospital stay at index admission |  |  |  |  | N/A |  | 0.007 |  | <0.001 |  | 0.088 |  | <0.001 |  | <0.001 |  | <0.001 |
| Yes | 262 | 22.9 | N/A | N/A |  | 12.6 |  | 5.6 |  | 2.3 |  | 18.7 |  | 24.4 |  | 9.9 |  |
| No | 880 | 77.1 | N/A | N/A |  | 7.3 |  | 29.4 |  | 0.8 |  | 8.8 |  | 5.1 |  | 3.2 |  |
| IMCU stay during index hospital admission |  |  |  |  | 0.197 |  | 0.279 |  | 0.322 |  | 0.594 |  | 0.600 |  | 0.480 |  | 0.255 |
| Yes | 76 | 6.7 | 9.0 | 28.9 |  | 11.8 |  | 14.5 |  | 1.3 |  | 9.2 |  | 11.8 |  | 1.3 |  |
| No | 1066 | 93.3 | 8.0 | 22.5 |  | 8.3 |  | 10.8 |  | 1.1 |  | 11.2 |  | 9.4 |  | 5.0 |  |
| Readmission to the ICU during index hospitalization |  |  |  |  | <0.001 |  | 0.722 |  | <0.001 |  | 0.269 |  | 0.109 |  | <0.001 |  | 1.000 |
| Yes | 27 | 2.4 | 34.0 | 81.5 |  | 3.7 |  | 55.6 |  | 3.7 |  | 22.2 |  | 40.7 |  | 3.7 |  |
| No | 1115 | 97.6 | 8.0 | 21.5 |  | 8.6 |  | 10.0 |  | 1.1 |  | 10.8 |  | 8.8 |  | 4.8 |  |
| Readmission to the IMCU during index hospitalization |  |  |  |  | <0.001 |  | 0.464 |  | <0.001 |  | 1.000 |  | 1.000 |  | 0.002 |  | 1.000 |
| Yes | 7 | 0.6 | 71.0 | 100 |  | 14.3 |  | 85.7 |  | 0.0 |  | 0.0 |  | 57.1 |  | 0.0 |  |
| No | 1135 | 99.4 | 8.0 | 22.5 |  | 8.5 |  | 10.6 |  | 1.1 |  | 11.1 |  | 9.3 |  | 4.8 |  |
| Preoperative event |  |  |  |  | 0.088 |  | 0.617 |  | 0.382 |  | N/A |  | 1.000 |  | 0.626 |  | 1.000 |
| Yes | 13 | 1.1 | 12.0 | 46.2 |  | 0.0 |  | 0.0 |  | N/A |  | 7.7 |  | 0.0 |  | 0.0 |  |
| No | 1129 | 98.9 | 8.0 | 22.7 |  | 8.6 |  | 11.2 |  | N/A |  | 11.1 |  | 9.7 |  | 4.8 |  |
| Intraoperative event |  |  |  |  | <0.001 |  | 0.564 |  | <0.001 |  | 1.000 |  | N/A |  | 0.055 |  | 0.072 |
| Yes | 126 | 11.0 | 10.0 | 38.9 |  | 7.1 |  | 19.8 |  | 0.8 |  | N/A |  | 14.3 |  | 7.9 |  |
| No | 1016 | 89.0 | 8.0 | 21.0 |  | 8.7 |  | 9.9 |  | 1.2 |  | N/A |  | 9.0 |  | 4.3 |  |
| Postoperative event within 90 days of index operation |  |  |  |  | <0.001 |  | <0.001 |  | <0.001 |  | 0.626 |  | 0.055 |  | N/A |  | 0.688 |
| Yes | 109 | 9.5 | 16.0 | 58.7 |  | 36.7 |  | 63.3 |  | 0.0 |  | 16.5 |  | N/A |  | 5.5 |  |
| No | 1033 | 90.5 | 8.0 | 19.2 |  | 5.5 |  | 11.0 |  | 1.3 |  | 10.5 |  | N/A |  | 4.6 |  |

**Table 3.** Outcomes of pediatric patients by age groups

|  | **No. of patients Total=1142** | | **LOS at Index Admission (in days)** | **Proportion of Patients with an Extended LOS** | **90-Day Unplanned Readmission Rate** | **90-Day Unplanned Reoperation Rate** | **Preoperative Event Rate** | **Intraoperative Event Rate** | **Postoperative Event Rate** | **90-Day Postoperative Mortality Rate** |
| --- | --- | --- | --- | --- | --- | --- | --- | --- | --- | --- |
| **Age Groups of Chidren** | **N** | **%** | **Median** | **%** | **%** | **%** | **%** | **%** | **%** | **%** |
| Neonatal period (From birth to under 28 days) | 12 | 1.1 | 55.5 | 83.3 | 33.3 | 50.0 | 0.0 | 8.3 | 33.3 | 25.0 |
| Infancy (28 days to under 12 months) | 32 | 2.8 | 9.0 | 28.1 | 6.3 | 15.6 | 0.0 | 18.8 | 6.3 | 0.0 |
| Toddler (12 months to under 2 years) | 8 | 0.7 | 10.0 | 37.5 | 12.5 | 0.0 | 0.0 | 0.0 | 0.0 | 0.0 |
| Early childhood (2 years to under 6 years) | 30 | 2.6 | 11.0 | 36.7 | 10.0 | 6.7 | 3.3 | 10.0 | 6.7 | 0.0 |
| Middle chidhood (6 years to under 12 years) | 39 | 3.4 | 11.0 | 33.3 | 12.8 | 17.9 | 5.1 | 20.5 | 5.1 | 2.6 |
| Adolescence (12 years to under 18 years) | 41 | 3.6 | 9.0 | 26.8 | 14.6 | 14.6 | 4.9 | 2.4 | 12.2 | 0.0 |

**Table 4.** Tumor histologies of the patients in the study cohort

| **Tumor Histology** |  | **N** | **%** |
| --- | --- | --- | --- |
| Anaplastic astrocytic glioma/Glioblastoma |  | 1 | 0.3 |
| Anaplastic astrocytoma |  | 8 | 2.2 |
| Anaplastic ependymoma |  | 1 | 0.3 |
| Anaplastic glioma |  | 1 | 0.3 |
| Anaplastic oligodendroglioma |  | 1 | 0.3 |
| Angiolipoma |  | 1 | 0.3 |
| Astroblastoma |  | 1 | 0.3 |
| Chondrosarcoma |  | 1 | 0.3 |
| Chordoma |  | 2 | 0.6 |
| Craniopharyngioma |  | 4 | 1.1 |
| Desmoplastic infantile astrocytoma |  | 1 | 0.3 |
| Desmoplastic infantile ganglioglioma |  | 1 | 0.3 |
| Diffuse astrocytic glioma |  | 1 | 0.3 |
| Diffuse astrocytoma |  | 6 | 1.6 |
| Diffuse glioma |  | 6 | 1.6 |
| Diffuse infiltrative glial tumor/Gliomatosis |  | 1 | 0.3 |
| Diffuse intrinsic pontine glioma |  | 3 | 0.8 |
| Diffuse leptomeningeal glioneuronal tumor |  | 1 | 0.3 |
| Dysembryoplastic neuroepithelial tumor |  | 4 | 1.1 |
| Embryonal tumors with multilayered rosettes |  | 1 | 0.3 |
| Eosinophilic granuloma |  | 1 | 0.3 |
| Ependymoma |  | 4 | 1.1 |
| Epithelioid and oncocytic tumor |  | 1 | 0.3 |
| Esthesioneuroblastoma |  | 2 | 0.5 |
| Ewing sarcoma |  | 1 | 0.3 |
| Ganglioglioma |  | 2 | 0.5 |
| Giant cell tumor |  | 1 | 0.3 |
| Glioblastoma |  | 51 | 13.9 |
| Glioma (Not further specified) |  | 2 | 0.5 |
| Hemangioblastoma |  | 6 | 1.6 |
| Hemangiopericytoma |  | 1 | 0.3 |
| High-grade astrocytoma with piloid features |  | 1 | 0.3 |
| Langerhans cell histiocytosis |  | 2 | 0.5 |
| Low grade glioneuronal tumor |  | 1 | 0.3 |
| Low-grade glioma |  | 2 | 0.5 |
| Medulloblastoma |  | 5 | 1.4 |
| Meningioma |  | 97 | 26.4 |
| Myxopapillary ependymoma |  | 1 | 0.3 |
| Neurinoma |  | 10 | 2.7 |
| Neurofibroma |  | 1 | 0.3 |
| Oligodendroglioma |  | 6 | 1.6 |
| Optic nerve glioma |  | 1 | 0.3 |
| Osteoma |  | 2 | 0.5 |
| Papillary tumor of pineal region |  | 1 | 0.3 |
| Pilocytic astrocytoma |  | 13 | 3.5 |
| Pituitary adenoma |  | 42 | 11.4 |
| Plasmacytoma |  | 1 | 0.3 |
| Pleomorphic adenoma |  | 1 | 0.3 |
| Rhabdomyosarcoma |  | 2 | 0.5 |
| Rosette-forming glioneuronal tumor |  | 2 | 0.5 |
| Solitary fibrous tumor/hemangiopericytoma |  | 1 | 0.3 |
| Subependymoma |  | 3 | 0.8 |
| Subependymoma and plexus papilloma |  | 1 | 0.3 |
| Tectal glioma |  | 2 | 0.5 |
| Trigeminal neuroma |  | 3 | 0.8 |
| Vestibular schwannoma |  | 48 | 13.0 |
| Xanthogranuloma |  | 1 | 0.3 |
| Total |  | 368 | 100.0 |

**Table 5.** WHO grade classification of tumors in the study cohort

| **WHO Grade** |  | **N** | **%** |
| --- | --- | --- | --- |
| I |  | 142 | 38.6 |
| II |  | 32 | 8.7 |
| III |  | 17 | 4.6 |
| IV |  | 58 | 15.8 |
| Unknown |  | 119 | 32.3 |
| Total |  | 368 | 100.0 |

**Table 6.** Origin of secondary tumors in the study cohort

| **Origin of secondary tumor** |  | **N** | **%** |
| --- | --- | --- | --- |
| Alveolar soft part sarcoma or PEComa |  | 1 | 1.4 |
| B-cell lymphoma |  | 5 | 6.9 |
| Breast cancer |  | 8 | 11.1 |
| Carcinoma of the cardia |  | 1 | 1.4 |
| Colon cancer |  | 2 | 2.8 |
| Esophageal cancer |  | 2 | 2.8 |
| Leptomeningeal melanomatosis |  | 1 | 1.4 |
| Lung cancer |  | 34 | 47.2 |
| MALT lymphoma |  | 2 | 2.8 |
| Melanoma |  | 4 | 5.6 |
| Merkel cell carcinoma |  | 1 | 1.4 |
| Neuroblastoma |  | 1 | 1.4 |
| Paranasal sinus cancer |  | 1 | 1.4 |
| Plasma cell myeloma |  | 1 | 1.4 |
| Prostate cancer |  | 1 | 1.4 |
| Rectal cancer |  | 2 | 2.8 |
| Renal cell carcinoma |  | 2 | 2.8 |
| Rhabdoid tumor |  | 1 | 1.4 |
| Undifferentiated metastatic carcinoma |  | 1 | 1.4 |
| Urachal cancer |  | 1 | 1.4 |
| Total |  | 72 | 100.0 |

| **Table 7.** Causes of unplanned reoperations  **Causes of Unplanned Reoperations** |  | **N** | **%** |
| --- | --- | --- | --- |
| Hydrocephalus |  | 50 | 25.00 |
| Postoperative hemorrhage |  | 21 | 10.50 |
| External ventricular drainage-associated complication |  | 18 | 9.00 |
| Elevated ICP/Cerebral edema/Midline shift/Herniation |  | 17 | 8.50 |
| Shunt-associated complication |  | 13 | 6.50 |
| Wound complication |  | 12 | 6.00 |
| Vasospasm |  | 9 | 4.50 |
| Intracranial pressure monitoring |  | 6 | 3.00 |
| Subcutaneous CSF accumulation |  | 5 | 2.50 |
| Control angiography secondary to complication |  | 4 | 2.00 |
| Ischemic infarction or transient ischemic attack |  | 4 | 2.00 |
| Cerebrospinal fluid fistula |  | 3 | 1.50 |
| Bone flap reimplantation secondary to unplanned osteoclastic decompressive craniotomy |  | 3 | 1.50 |
| Recurrence of intracranial abscess |  | 2 | 1.00 |
| Epistaxis |  | 2 | 1.00 |
| Residual hematoma |  | 2 | 1.00 |
| Metastasis |  | 2 | 1.00 |
| Spinal stenosis |  | 2 | 1.00 |
| Tumor progression |  | 2 | 1.00 |
| Recurrence of spinal disc herniation |  | 2 | 1.00 |
| Intracranial abscess |  | 2 | 1.00 |
| Ommaya reservoir-associated complication |  | 2 | 1.00 |
| Pneumocephalus |  | 2 | 1.00 |
| Second-look angiography |  | 1 | 0.50 |
| Recurrence of spinal disc herniation with postoperative hemorrhage |  | 1 | 0.50 |
| Progression of trigeminal neuralgia |  | 1 | 0.50 |
| Residual tumor |  | 1 | 0.50 |
| Inconclusive biopsy |  | 1 | 0.50 |
| Inconclusive epilepsy monitoring |  | 1 | 0.50 |
| Accumulation of cerebrospinal fluid |  | 1 | 0.50 |
| Progression of intracerebral hemorrhage |  | 1 | 0.50 |
| Perforation of carotid artery |  | 1 | 0.50 |
| Abscess progression |  | 1 | 0.50 |
| Newly detected lesion of unknown origin |  | 1 | 0.50 |
| Miscellaneous |  | 4 | 2.00 |

**Table 8.** Preoperative events

| **Preoperative Events** | **N** | **%** |
| --- | --- | --- |
| Non-central nervous system infection | 5 | 33.3 |
| Seizure | 2 | 13.3 |
| Hypertension | 2 | 13.3 |
| Pneumothorax | 1 | 6.7 |
| Deep vein thrombosis | 1 | 6.7 |
| Acute kidney failure | 1 | 6.7 |
| Coagulopathy | 1 | 6.7 |
| Syncope | 1 | 6.7 |
| Accidental fall resulting in physical injury | 1 | 6.7 |
| Total | 15 | 100.0 |

**Table 9.** Intraoperative events

| **Intraoperative Events** | **N** | **%** |
| --- | --- | --- |
| Surgical intraoperative events |  |  |
| Hemorrhage | 21 | 13.8 |
| Dural laceration | 11 | 7.2 |
| Coagulopathy | 8 | 5.3 |
| Seizure | 3 | 2.0 |
| Cerebrospinal fluid leakage | 3 | 2.0 |
| Failure to preserve the nasal conchae during transsphenoidal surgery | 3 | 2.0 |
| Difficulty placing the extraventricular drainage or lumbar drainage | 2 | 1.3 |
| Incomplete resection of disc herniation due to calcification | 2 | 1.3 |
| Failure to evacuate intracerebral hematoma | 2 | 1.3 |
| Scalp laceration secondary to use of a Mayfield head clamp | 1 | 0.7 |
| Perforation of carotid artery | 1 | 0.7 |
| Perforation of the superior branch of anterior choroidal artery | 1 | 0.7 |
| Injury of the olfactory nerve during resection of a meningioma | 1 | 0.7 |
| Failure to obtain a tumor sample suitable for histological exam | 1 | 0.7 |
| Cerebral edema | 1 | 0.7 |
| Cerebellar edema | 1 | 0.7 |
| Difficulty placing the catheter of Ommaya reservoir | 1 | 0.7 |
| Difficulty placing shunt | 1 | 0.7 |
| Failure to remove peritoneal catheter | 1 | 0.7 |
| Failure to remove ventricular catheter | 1 | 0.7 |
| Failure to place depth electrodes | 1 | 0.7 |
| Accidental cutting of depth electrode | 1 | 0.7 |
| Displacement of reference electrode | 1 | 0.7 |
| Difficulty placing depth electrodes | 1 | 0.7 |
| Failed attempt at lumbar puncture | 1 | 0.7 |
|  |  |  |
| Thromboembolic events | 5 | 3.3 |
| Difficulty to puncture the femoral artery | 5 | 3.3 |
| Difficulty placing the guiding catheter | 5 | 3.3 |
| Residual aneurysm neck after clipping or coiling | 4 | 2.6 |
| Residual perfusion of the aneurysm after coiling | 4 | 2.6 |
| Difficulty placing coils into the aneurysm | 3 | 2.0 |
| Extraaneurysmal dissolution of coil | 1 | 0.7 |
| Anesthesiologic intraoperative events |  |  |
| Asystole | 7 | 4.6 |
| Hemodynamic instability | 5 | 3.3 |
| Air embolism | 4 | 2.6 |
| Bradycardia | 4 | 2.6 |
| Difficult airway | 3 | 2.0 |
| Hypertension | 3 | 2.0 |
| Hypotension | 2 | 1.3 |
| Failure to place permanent urinary catheter | 2 | 1.3 |
| Pneumothorax | 1 | 0.7 |
| Quincke's edema | 1 | 0.7 |
| Paravenous injection through peripheral venous catheter | 1 | 0.7 |
| Difficulty placing arterial catheter | 1 | 0.7 |
| Accidental extubation | 1 | 0.7 |
| Pain | 1 | 0.7 |
| Panic attack | 1 | 0.7 |
| Technical equipment-related intraoperative events |  |  |
| Failures in equipment and technological devices | 11 | 7.2 |
| Missing equipment or technical device in the operating theatre | 6 | 3.9 |
| Total | 152 | 100.0 |

**Table 10.** Categories and definitions of postoperative events

| **New postoperative neurologic deficit:** Emergence of a focal neurologic deficit that was not present preoperatively and that resulted in a prolonged hospitalization, readmission, or reoperation. Neurologic deficits caused by the progression of the underlying disease were excluded. Moreover, any sudden neurological deterioration which could be explained by a cerebrovascular event such as ischemia or intracranial hemorrhage was also not included. |
| --- |
| **Postoperative hemorrhage:** Postoperative bleeding that required a reoperation or resulted in death. Patients who received antithrombotic therapy for prophylaxis prior to the index surgery were excluded. Further, non-elective patients who presented with an intracranial hemorrhage that later experienced a further bleeding are not included. |
| **Ischemic infarction:** Any radiologically verified postoperative ischemic event that was not caused by the underlying disease of the patient. |
| **Central nervous system infection:** Microbiologically confirmed or clinically proven infection of the central nervous system including meningitis, encephalitis, ventriculitis, abscess, or empyema. |
| **Subcutaneous cerebrospinal fluid accumulation:** Presence of an excessive accumulation of cerebrospinal fluid under the skin that resulted in a prolonged hospitalization, readmission, or reoperation. |
| **Cerebrospinal fluid fistula and/or leakage:** Cerebrospinal fluid fistula and/or leakage requiring treatment and resulting in a prolonged hospitalization, readmission, or reoperation. |
| **Malfunction, disconnection, or dislocation of an implanted device:** Ventriculoperitoneal shunt-, lumbar peritoneal shunt-, subdural-peritoneal shunt-, external ventricular drain-, lumbar drain- or Ommaya reservoir-associated adverse events that resulted in a prolonged hospitalization, readmission, or reoperation. |
| **Wound complications:** Wound disruption/dehiscence, surgical site infection, significant hematoma formation in wound area, wound healing impairment, or necrosis of the wound that resulted in a prolonged hospitalization, readmission, or reoperation. |
| **Pneumocephalus:** Radiologically proven presence of air in the intracranial space after index surgery that resulted in a prolonged hospitalization, readmission, or reoperation. |
| **Pituitary surgery associated events:** Syndrome of inappropriate antidiuretic hormone secretion, diabetes insipidus, epistaxis, and electrolyte imbalances that resulted in a prolonged hospitalization, readmission, or reoperation. |

**Table 11.** Postoperative events

| **Postoperative Events** | **Elective Cases** | **Non-elective Cases** | **Total** | **%** |
| --- | --- | --- | --- | --- |
| Malfunction, disconnection, or dislocation of an implanted device | 6 | 13 | 24 | 17.9 |
| Wound complications | 13 | 7 | 20 | 14.9 |
| New postoperative neurologic deficit | 11 | 6 | 17 | 12.7 |
| Central nervous system infection | 4 | 10 | 14 | 10.4 |
| Ischemic infarction | 9 | 4 | 14 | 10.4 |
| Pituitary surgery associated events | 14 | 0 | 14 | 10.4 |
| Postoperative hemorrhage | 8 | 3 | 11 | 8.2 |
| Subcutaneous cerebrospinal fluid accumulation | 7 | 2 | 9 | 6.7 |
| Cerebrospinal fluid fistula and/or leakage | 4 | 3 | 7 | 5.2 |
| Pneumocephalus | 3 | 1 | 4 | 3.0 |
| Total | 79 | 55 | 134 | 100.0 |

**Table 12.** Results of the multivariate logistic regression models for perioperative events

|  | **Variable** | ***P*** | **Odds Ratio** | **95% Confidence Interval** |
| --- | --- | --- | --- | --- |
| **Preoperative Events** | Preoperative sodium of <135 mmol/L | 0.042 | 575.70 | 1.26-262414.91 |
|  | Preoperative C-reactive protein of ≥0,5 mg/dL | 0.012 | 227.76 | 3.29-15783.43 |
| **Intraoperative Events** |  |  |  |  |
|  | Presence of coronary heart disease | 0.024 | 2.77 | 1.15-6.72 |
|  | Preoperative use of angiotensin II receptor blockers | 0.030 | 1.93 | 1.06-3.49 |
| **Postoperative Events** | Cohort ‘pituitary surgery’ | <0.001 | 22.38 | 6.19-80.91 |
|  | Cohort ‘Hyhrocephalus’ | 0.008 | 8.03 | 1.72-37.47 |
|  | Presence of coagulopathy | <0.009 | .8.90 | 1.74-45.57 |
|  | Number of neurosurgical operations during index hospitalization equal to 2 | <0.001 | 5.79 | 2.60-12.94 |
|  | Number of neurosurgical operations during index hospitalization equal to 3 or more | <0.001 | 24.83 | 7.17-86.01 |

**Figures**

**Fig 1.** Study population by cohort


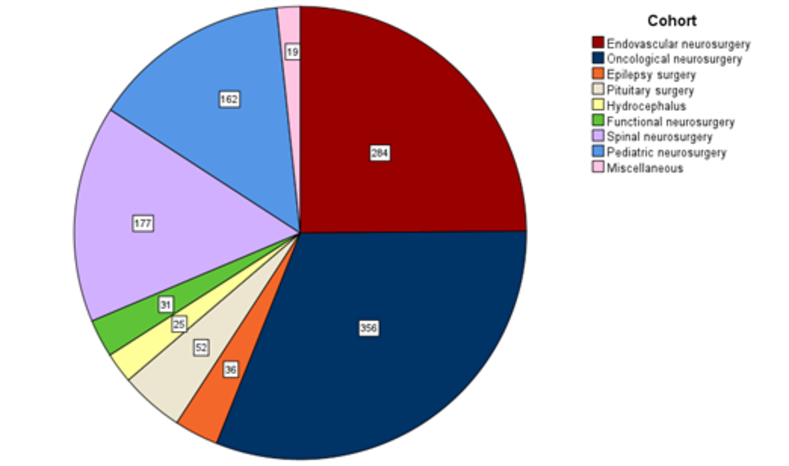


**Fig 2.** Time until reoperations after index surgery


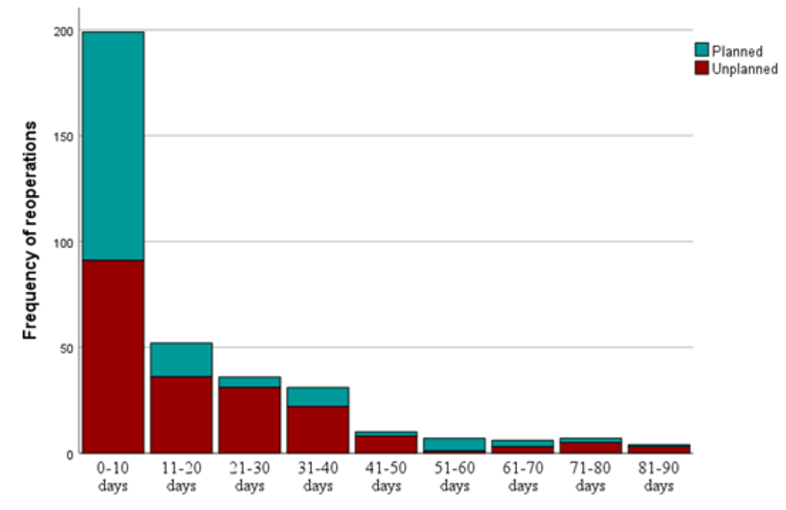


**Fig 3.** Kaplan-Meier curve depicting the time to death after discharge from index admission stratified by extended length of hospital stay

**
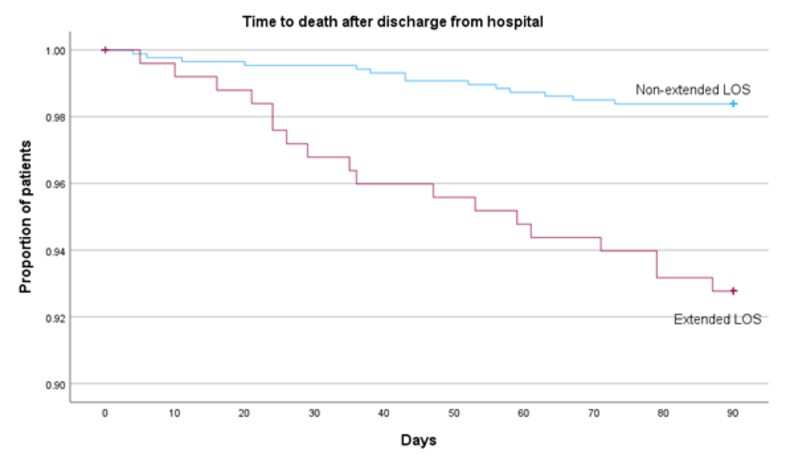
**

**Fig 4.** Kaplan-Meier curve depicting the time to death after discharge from index hospital stay stratified by 90-day readmission


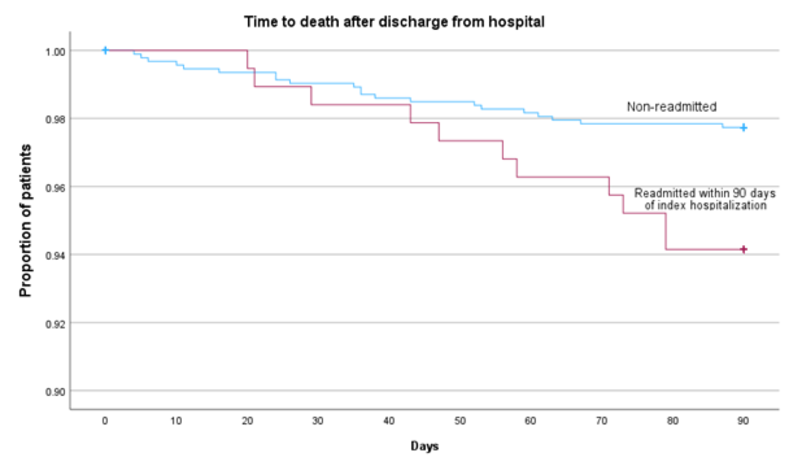


**Fig 5.** Kaplan-Meier curve depicting the time to death after index operation stratified by 90-day reoperation

**
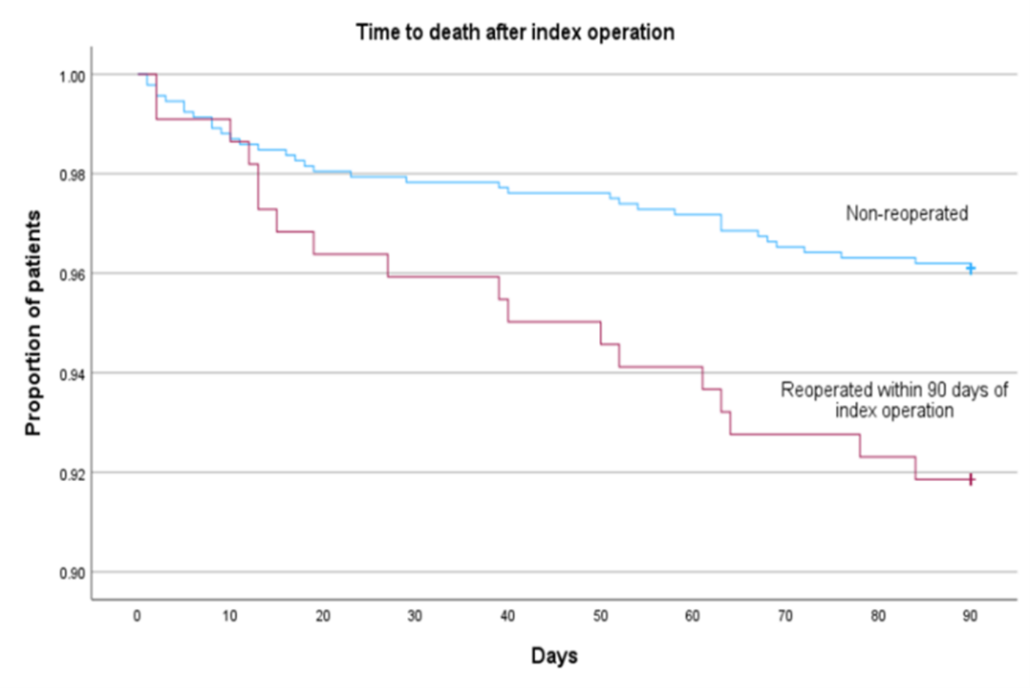
**
